# Phosphoproteomic Profiling Reveals Overlapping and Distinct Signaling Pathways in *Dictyostelium discoideum* in Response to Two Different Chemorepellents

**DOI:** 10.3390/cells15010060

**Published:** 2025-12-29

**Authors:** Salman Zahir Uddin, Ramesh Rijal, Darrell Pilling, Richard H. Gomer

**Affiliations:** 1 Department of Biology, Texas A&M University, ILSB 301 Old Main Drive, College Station, TX 77843-3474, USA; szuddin@tamu.edu (S.Z.U.); ramesh.rijal@usm.edu (R.R.); dpilling@bio.tamu.edu (D.P.); 2 School of Biological, Environmental, and Earth Sciences, University of Southern Mississippi, Hattiesburg, MS 39406, USA

**Keywords:** proteomics, phosphoproteomics, *Dictyostelium discoideum*, chemorepulsion, small GTPases, AprA, polyphosphate

## Abstract

Chemorepulsion mechanisms for eukaryotic cells are poorly understood. We performed proteomics and phosphoproteomics to elucidate how *Dictyostelium discoideum* responds to its two endogenous chemorepellent signals, the protein AprA and inorganic polyphosphate (polyP). AprA and polyP affected levels of more than 200 proteins, with an overlap of both upregulating 25 proteins and downregulating two proteins. Two proteins were upregulated by AprA but downregulated by polyP, while two others showed the opposite trend. Surprisingly, many of the AprA- and polyP-regulated proteins are associated with RNA metabolism and ribosomes. AprA increased phosphorylation of 15 proteins and decreased phosphorylation of 36 proteins. PolyP increased phosphorylation of 12 proteins and decreased phosphorylation of 12 proteins. As expected, the two chemorepellents affected phosphorylation of signal transduction/ motility proteins, but unexpectedly affected phosphorylation of RNA-associated proteins. Both AprA and polyP decreased phosphorylation of five proteins including the Ras-interacting protein RipA and guanine nucleotide exchange factors (GEFs) such as the RacGEF GxcT. Mutants lacking RipA or GxcT were unresponsive to both AprA and polyP chemorepulsion. Together, this work supports the idea that rather than activating the same chemorepulsion mechanism, AprA and polyP activate only partially overlapping chemorepulsion mechanisms, and identifies two new components that are used by both chemorepellents.

## 1. Introduction

Directed cell migration is a fundamental biological process essential for morphogenesis, tissue repair, and immune surveillance [1,2]. While the molecular mechanisms of chemoattraction, the process by which cells migrate toward stimuli, have been extensively studied, the opposite behavior, chemorepulsion, remains comparatively underexplored [2,3]. Chemorepulsion enables cells to migrate away from harmful or crowded environments and plays vital roles in immune regulation, inflammation resolution, and spatial patterning during development [4,5].

The social amoeba *D. discoideum* is a well-established eukaryotic model organism for investigating chemotaxis due to its genetic tractability and the conservation of many signaling pathways with higher eukaryotes [6,7]. In this system, two endogenous chemorepellents have been identified: AprA, a ~60 kDa secreted autocrine protein, and inorganic polyphosphate (polyP), a polymer of phosphate [8,9,10,11,12]. The two repellents can function independently, but both cause cells at the edge of a colony to move away from the colony, presumably to find sources of food. In *D. discoideum* and other systems, a localized activation of Ras at one sector of the cell membrane activates pseudopod formation and movement in the direction of the pseudopod [13,14,15]. A gradient of AprA induces repulsion by inhibiting Ras activation and pseudopod formation at the side of the cell closest to the source of AprA, causing the cell to move in any direction except toward the source of AprA [10,11]. In contrast, a gradient of polyP induces chemorepulsion by activating Ras at multiple cortical sites and increasing pseudopod formation, particularly at the side of the cell furthest from the polyP source [12].

Although both AprA and polyP can trigger repulsion, their signaling mechanisms only partially overlap. AprA signals through the G protein-coupled receptor (GPCR) GrlH, activating the heterotrimeric G-protein subunits Gα8 and Gβ, which in turn signal through PakD, CnrN, TORC2, Erk1, and the Ras proteins RasC and RasG [8,9,10,16]. PolyP signals through the GPCR GrlD, using different G-protein subunits, and uses PI3 kinases, phospholipase C, and effectors such as WasA and NapA to control actin dynamics [12,17,18]. While AprA requires both RasC and RasG for repulsion, polyP requires RasC but not RasG, underscoring their differences in small GTPase regulation despite sharing other core signaling elements [12].

Extracellular levels of AprA rise as cultures transition from low to high cell density and nutrients begin to decline [19]. PolyP likewise accumulates extracellularly as cultures reach high cell density or become nutrient-limited [20], positioning both molecules as components of a broader density-sensing system that integrates environmental conditions with downstream cytoskeletal and signaling responses. Both AprA and polyP inhibit cell proliferation [20,21]. AprA-mediated proliferation inhibition involves RasC/RasG and Erk1 signaling pathway components also required for chemorepulsion, but requires some signal transduction pathway components not needed for repulsion to inhibit proliferation [4,22,23]. PolyP also uses some components needed for repulsion to also inhibit proliferation, and has at least one pathway component not needed for repulsion that is used to inhibit proliferation [12,17,18].

At the population level, the repulsion induced by AprA and polyP contributes to colony organization during growth and resource depletion. Elevated levels of these extracellular cues promote outward dispersal from dense, nutrient-depleted regions, a behavior consistent with ecological bet-hedging, in which a subset of cells commits to development while others migrate to explore new nutrient sources [24]. This ecological role aligns with the proliferation suppression and translational adjustments observed downstream of AprA and polyP.

Many extracellular signals affect phosphorylation of specific proteins [5,25,26]. Our understanding of chemorepulsion pathway components in *D. discoideum* only used known mutants. To gain a broader perspective, in this study we used proteomics and phosphoproteomics to elucidate the effects of AprA and polyP on cells. We find that AprA and polyP affect levels and phosphorylation of partially overlapping pathway components centered on small GTPase signaling and show that two proteins whose phosphorylation is reduced by AprA and polyP are necessary for the effects of both chemorepellents on repulsion but not proliferation.

## 2. Materials and Methods

### 2.1. Cell Strains and Culture

*D. discoideum* strains Ax2 (Dictybase identifier DBS0237699) [27] and KAx3 (DBS0236487) [28] were obtained from the *Dictyostelium* stock center [29]. The strains *ripA*^−^ (DBS0236900) [30] and *gxcT*^−^ (DBS0350268) [31] were gifts from Dr. Alan Kimmel (Laboratory of Cellular and Molecular Biology, NCI, NIH, Bethesda, MD, USA) and Dr. Miho Iijima (Department of Cell Biology, Johns Hopkins University School of Medicine, Baltimore, MD, USA), respectively. Cells were cloned on SM/5 agar with DB *Escherichia coli* (DBS0350636, DictyBase, Northwestern University, Chicago, IL, USA) and then grown in HL5 medium (#HLG0101, Formedium, Hunstanton, UK) containing 100 µg/mL dihydrostreptomycin (#S-150-50, GoldBio, St. Louis, MO, USA) and 100 µg/mL ampicillin (#A-301-25, GoldBio) as previously described [32,33]. For HL5 growth, cells were either in submerged culture on 10 cm tissue culture dishes (#10861-680, Avantor, Randor, PA, USA) or in suspension at 22 °C with continuous shaking at 180 rpm [27,32]. Mutants were grown under constant selection with 5 µg/mL blasticidin (#B-800-25, GoldBio) in both submerged and shaking culture.

### 2.2. Stimulation with AprA and polyP

Ax2 cells were grown in 50 mL of HL5 medium at 22 °C with shaking at 180 rpm until mid-log phase (3 × 10^6^ cells/mL) [27,32]. Cells were collected by centrifugation at 380× *g* for 5 min at 22 °C and washed twice by resuspension in 10 mL of HL5 and centrifugation. Cells were then resuspended in 10 mL of HL5 to 1 × 10^6^ cells/mL. For AprA treatment, recombinant AprA (rAprA) was expressed and purified as previously described [11,34] and concentrated using a 10 kDa molecular-weight-cutoff Spin-X UF 20 centrifugal concentrator (#431488; Corning, Corning, NY, USA). The concentrated protein was filter sterilized through a PALL Acrodisc 0.2 µm Supor membrane syringe filter (#4602; Cytiva, Marlborough, MA, USA) and stored at 4 °C. After filtration, the protein concentration was determined by densitometry on SDS-PAGE using bovine serum albumin (BSA) as a standard (Supplementary Figure S1). For cell stimulation, purified rAprA was then diluted in HL5 medium to a final concentration of 300 ng/mL, a dose previously shown to elicit robust and reproducible chemorepulsion responses [8,11,21]. For polyphosphate (polyP) stimulation, sodium polyphosphate (average chain length ~46; #S0169, Spectrum Chemical, New Brunswick, NJ, USA) was freshly prepared as a 70 mg/mL stock in water and added to a final concentration of 210 μg/mL, a concentration previously demonstrated to induce chemorepulsion in *Dictyostelium* [12,20]. The sodium polyphosphate used in this study had an average chain length of approximately 46 phosphate residues, consistent with the size range of endogenous *Dictyostelium* polyP (~50 residues) determined by PAGE analysis [35] and closely matching the ~40-mer extracellular polyP detected in conditioned medium from starved cells [20]. 3.5 mL aliquots of unstimulated cells (0 min) and cells after stimulation with AprA or polyP at 10, 30, or 60 min were transferred into pre-chilled 15 mL conical tubes (89039-664, VWR, Radnor, PA, USA), collected by centrifugation at 2000× *g* for 5 min at 4 °C, and placed on ice. Pellets were resuspended in 150 µL of ice-cold RIPA buffer (#PI89901, Thermo Fisher Scientific, Waltham, MA, USA) supplemented with phosphatase inhibitor cocktail (#78420, Thermo Fisher Scientific, Waltham, MA, USA) and complete protease inhibitor cocktail (#11836170001, Roche Diagnostics, Basel, Switzerland). Lysates were incubated on ice for 30 min with occasional mixing to enhance extraction efficiency. Following centrifugation at 18,000× *g* for 10 min at 4 °C, 140 µL of the clarified supernatants were transferred to pre-chilled Eppendorf tubes. 10 μL aliquots were set aside for protein concentration determination and the remaining supernatant was flash-frozen in liquid nitrogen and stored at −140 °C [32,36].

### 2.3. Proteomic and Phosphoproteomic Sample Preparation and Analysis

Proteomics and phosphoproteomics were carried out at the University of Texas Southwestern (UTSW) Proteomics Core Facility (Dallas, TX, USA). Samples were shipped on dry ice to UTSW, where all sample processing, including protein quantification, reduction and alkylation, enzymatic digestion, phosphopeptide enrichment using titanium dioxide (TiO_2_) beads, and peptide desalting, was performed following their standard protocols [37,38,39]. LC-MS/MS analysis was performed on an Orbitrap Fusion mass spectrometer (Thermo Fisher Scientific, Waltham, MA, USA) using standard gradient methods. Raw data were processed and searched against the *D. discoideum* UniProt protein database using MaxQuant software (v1.6.17), applying a 1% false discovery rate at peptide and protein levels [40,41]. Raw and processed proteomic data was uploaded to MassIVE at the University of California at San Diego Center for Computational Mass Spectrometry with accession number MSV000098894.

### 2.4. Data Normalization and Analysis

Protein and phosphoprotein abundances were normalized within each replicate by dividing each individual abundance value by the total protein or phosphoprotein abundances of that replicate, thereby controlling for differences in sample loading and instrument response for both the total proteome and the phosphoproteome [42]. Fold changes were then calculated as the ratio of normalized abundance at 10, 30, and 60 min relative to the normalized abundance at 0 min for the same protein or phosphoprotein. Proteins or phosphoproteins with missing values in one or more replicates were excluded from analysis and reported separately in Supplementary Files S1–S4. This conservative filtering reduced the dataset by <1% (12 proteins for AprA, and 36 for polyP). Proteins or phosphoproteins with zero abundance in all three replicates at the 0 min time point were excluded from fold-change and statistical analyses; we identified two such proteins under polyP treatment and one under AprA treatment across both the time points, which are reported separately in the Results. Apart from these explicitly reported cases, all other proteins and phosphoproteins were included in the downstream analyses. Differential protein and phosphoprotein abundance were assessed using paired two-tailed Student’s *t*-tests applied to normalized replicate values. Significance thresholds in the main text refer to nominal *p*-values (*p* < 0.05). To provide transparency regarding multiple hypothesis testing across the 5383 and 5015 quantified proteins for AprA and polyP, respectively, we additionally computed Benjamini–Hochberg false discovery rate (FDR)–adjusted *p*-values for all proteins and phosphoproteins. An FDR threshold of 1% (q < 0.01) was applied, and proteins meeting this criterion are reported in Supplementary Files S1–S4. Because discovery proteomics studies with *n* = 3 replicates often have limited power under stringent FDR thresholds, proteins meeting nominal *p* < 0.05 are reported as exploratory candidates, while corresponding FDR values (q values) allow readers to assess the likelihood of false discovery. Volcano plots were generated using Prism 10.4.0 (GraphPad Software, San Diego, CA, USA). Functional enrichment analysis of significant proteins and phosphoproteins was performed using ShinyGO v0.85 [43] using database *Dictyostelium discoideum* STRINGv12.0 (STRING.44689.Dictyostelium, ID#44689) with parameters set to an FDR cutoff of 0.05, a pathway size range of 5–1000 genes, and a maximum of 30–40 pathways displayed. Gene Ontology (GO) terms and SMART domain annotation [44] were included in the enrichment workflow.

### 2.5. Protein Annotation Using AlphaFold and Foldseek

To assign putative functions to uncharacterized protein hits identified in the proteomic and phosphoproteomic analyses, we conducted sequence similarity searches using the BLAST tool available through the NCBI web interface (https://blast.ncbi.nlm.nih.gov/Blast.cgi, accessed on 20 October 2025), followed by structural homology searches using AlphaFold (https://alphafold.ebi.ac.uk, accessed on 20 October 2025) [45,46] and Foldseek (https://search.foldseek.com, accessed on 20 October 2025) [47]. For both AprA- and polyP-treated samples, only proteins exhibiting greater than twofold changes in abundance or phosphorylation were selected for characterization. We also used Alphafold/Foldseek to characterize 6 proteins whose phosphorylation was affected by both AprA and polyP.

### 2.6. Functional Validation Assays

Chemorepulsion assays were performed in Insall chambers as previously described [8,10,48]. HL5 medium was added to the central well, while either 300 ng/mL AprA or 210 µg/mL polyP diluted in HL5 was placed in the outer well, with an equal volume of HL5 media used as a control. Time-lapse images were collected every 15 s for 60 min using an inverted microscope. Migration parameters including forward migration index (FMI), speed, and persistence were quantified using the ibidi Chemotaxis and Migration Tool 2.0 (Stand-alone application; ibidi GmbH, Gräfelfing, Germany).

Proliferation inhibition assays were performed as described [16], with cells cultured at 5 × 10^5^ cells/mL in HL5 medium and treated with or without 300 ng/mL AprA or 700 µg/mL polyP. Viable cell counts were determined at 24 h using a hemocytometer, and proliferation was calculated as a percentage of the untreated control for each replicate.

### 2.7. Statistical Analysis

Statistical analyses were performed using Microsoft Excel or Prism 10.4.0 (GraphPad Software, Boston, MA, USA).

## 3. Results

### 3.1. AprA and polyP Have Mostly Different Effects on Protein Levels and Phosphorylation

**Figure 1:**
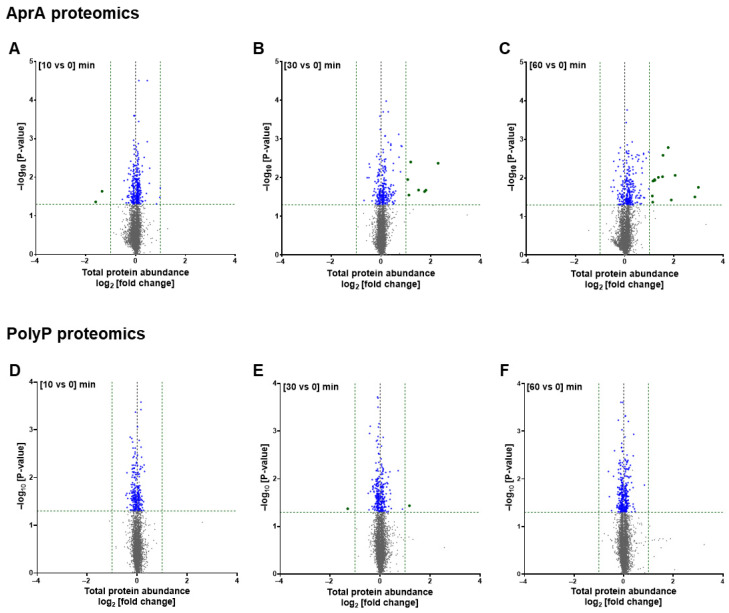
Comparative analysis of proteomic changes induced by AprA and polyP. Volcano plots of protein abundance changes at 10, 30 and 60 min post stimulation (*x*-axis: log_2_ [fold change]; *y*-axis: −log_10_ [*p*-value]). (**A**–**C**) Proteomic changes following AprA stimulation at (**A**) 10, (**B**) 30, and (**C**) 60 min (*n* = 5383 proteins). (**D**–**F**) Proteomic changes following polyP stimulation at (**D**) 10, (**E**) 30, and (**F**) 60 min (*n* = 5015 proteins). Green vertical dashed lines mark the significance threshold (log_2_ fold change = ±1, equivalent to a ≥2-fold change in abundance), while the horizontal dashed line marks the statistical significance cutoff (*p* < 0.05). Green dots indicate proteins with |log_2_ fold change| ≥ 1 and *p* < 0.05; blue dots indicate proteins with |log_2_ fold change| < 1 and *p* < 0.05; gray dots indicate non-significant proteins.

To investigate how AprA and polyP modulate signaling in *D. discoideum*, we first assessed effects on the proteome of proliferating cells. Out of 5383 proteins quantified (Supplementary File S1), at 10 min, AprA increased levels of 184 and decreased levels of 106 proteins, including 2 proteins (glucanase and nuclear speckle splicing regulatory protein 1 N-terminal domain-containing protein) with more than a 2-fold decrease (Figure 1A; Supplementary File S1, Tabs 3 and 6). At 30 min AprA increased levels of 163 proteins including 7 (Figure 1B; Supplementary File S1, Tabs 4 and 6) with more than 2-fold increases and decreased levels of 81 proteins. At 60 min AprA increased levels of 211 proteins, including 13 (Figure 1C; Supplementary File S1, Tabs 5 and 6) with more than 2-fold increases and decreased levels of 57 proteins. A previously uncharacterized protein (Q550S3) was upregulated by more than 2-fold at 60 min by AprA. Annotation by BLAST, AlphaFold, and FoldSeek suggested strong similarity to PhoPQ-activated pathogenicity-related proteins and to AprA-like isoforms (Supplementary File S1, Tab 6). Proteins meeting nominal *p* < 0.05 are reported as exploratory observations; corresponding Benjamini–Hochberg FDR–adjusted protein candidates are provided in Supplementary File S1, Tab 7. Proteins with a missing value in a replicate were excluded from analysis and are listed in Supplementary File S1, Tab 8. One protein (MAP3 kinase 12 inhibitory protein); had zero abundance in all three replicates at 0 min but was detectable at 60 min.

Out of 5015 proteins quantified (Supplementary File S2), at 10 min, polyP upregulated levels of 97 proteins and downregulated 142 proteins; at 30 min upregulated 118 proteins including one (G domain-containing protein) increased by more than 2-fold and downregulated 146 proteins including one (prespore protein MF12) decreased by more than 2-fold; and at 60 min upregulated 152 proteins and downregulated 168 proteins (Figure 1D–F; Supplementary File S2, Tabs 2–6). Proteins meeting nominal *p* < 0.05 are discussed in this section; corresponding Benjamini–Hochberg FDR-adjusted proteins are provided in Supplementary File S2, Tab 7. Two proteins had zero abundance in all three replicates at 0 min (Methyltransferase type 12 domain-containing protein and SAM domain-containing protein), were excluded from fold-change and statistical calculations but are listed in Supplementary File S2, Tab 8.

**Figure 2:**
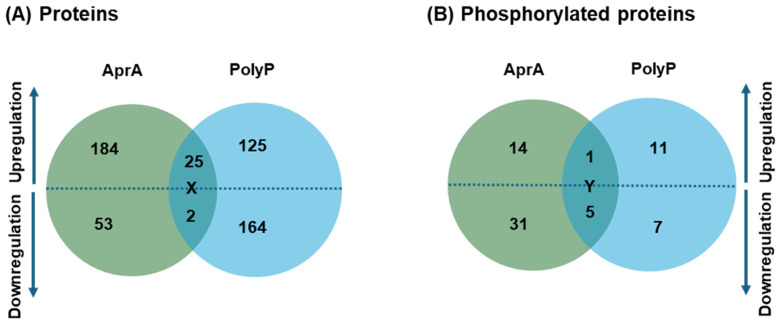
Proteins and phosphoproteins regulated by AprA and polyP. (**A**) Venn diagram of significantly regulated proteins after 60 min of AprA or polyP treatment. X indicates two proteins upregulated in AprA but downregulated in polyP, two proteins downregulated in AprA and upregulated in polyP, two proteins downregulated in both, and 25 proteins upregulated in both. (**B**) Venn diagram of significantly regulated phosphoproteins whose phosphorylation changes significantly relative to their total protein abundance at 60 min. Y indicates upregulation of one phosphoprotein, and downregulation of five phosphoproteins in both treatments. Numbers in each diagram represent proteins, or phosphoproteins that are significantly upregulated (above dashed line) or downregulated (below dashed line).

At 60 min, comparison of AprA- and polyP-treated proteomes revealed 25 proteins which were upregulated by both treatments, two (a small GTPase and a transmembrane protein) were downregulated by both, two (an ubiquitin carboxyl-terminal hydrolase and a B box-type domain-containing protein) were upregulated by AprA but downregulated by polyP, and two (a non-specific serine/threonine protein kinase and a FNIP/ankyrin repeat-containing protein) were downregulated by AprA but upregulated by polyP (Figure 2A; Supplementary File S7, Tab 2). For both AprA and polyP, many proteins did not exhibit significant changes, indicating a targeted rather than a global response.

**Figure 3:**
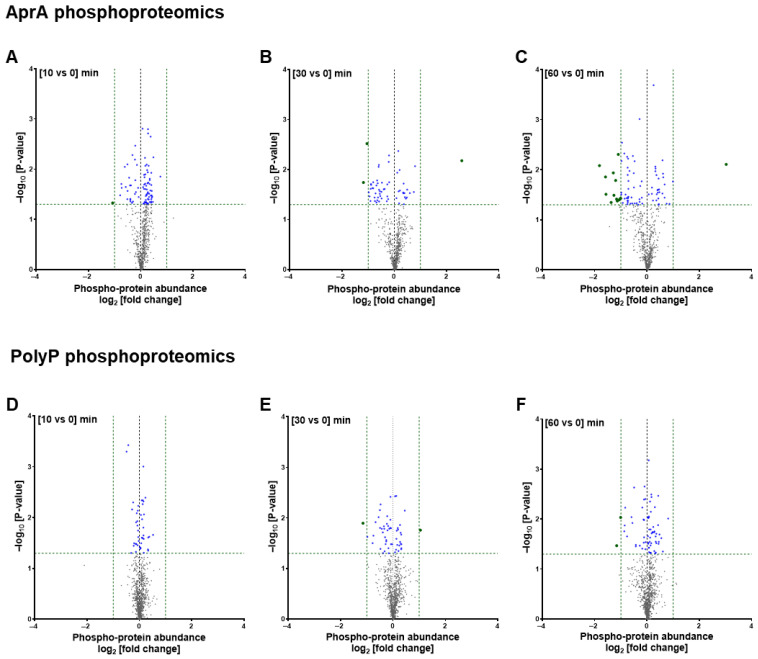
Comparative analysis of phosphoproteomic changes induced by AprA and polyP. Volcano plots of phosphoprotein abundance changes at 10, 30 and 60 min post stimulation (*x*-axis: log_2_ [fold change]; *y*-axis: −log_10_ [*p*-value]). (**A**–**C**) Phosphoproteomic changes following AprA treatment at (**A**) 10, (**B**) 30, and (**C**) 60 min (*n* = 602 phosphoproteins). (**D**–**F**) Phosphoproteomic changes following polyP treatment at (**D**) 10, (**E**) 30, and (**F**) 60 min (*n* = 846 phosphoproteins). Green vertical dashed lines mark the significance threshold (log_2_ fold change = ±1, equivalent to a ≥2-fold change in abundance), while the horizontal dashed line marks the statistical significance cutoff (*p* < 0.05). Green dots indicate proteins with |log_2_ fold change| ≥ 1 and *p* < 0.05; blue dots indicate proteins with |log_2_ fold change| < 1 and *p* < 0.05; gray dots indicate non-significant proteins.

Phosphoproteomics profiling of the AprA-treated samples showed that among 602 quantified phosphoproteins, at 10 min, AprA increased levels of 60 phosphoproteins and decreased levels of 26 phosphoproteins including one (hypothetical, similar to *Entamoeba histolytica* 20 kDa antigen) downregulated by more than 2-fold (Figure 3A; Supplementary File S3, Tabs 3 and 6). At 30 min, AprA increased 20 phosphoproteins including one (RRM domain-containing protein) upregulated by more than 2-fold and decreased 39 phosphoproteins including 2 downregulated by more than 2-fold (Figure 3B; Supplementary File S3, Tabs 4 and 6). At 60 min AprA increased levels of 30 phosphoproteins including one (RRM domain-containing protein) with more than 2-fold upregulation and decreased 53 phosphoproteins including 13 downregulated by more than 2-fold (Figure 3C; Supplementary File S3, Tabs 5 and 6). One phosphoprotein (Calmodulin), with missing values in all the replicates at one or all time points, was excluded from fold-change and statistical calculations but mentioned in the Supplementary File separately (Supplementary File S3, Tab 8). At 10 min, polyP increased levels of 24 phosphoproteins and decreased 21 phosphoproteins; at 30 min, increased 21 phosphoproteins including one (Ubiquitin-like domain-containing protein) upregulated by more than 2-fold and decreased 33 phosphoproteins including one (Rac guanine nucleotide exchange factor T) downregulated by more than 2-fold; and at 60 min, polyP increased levels of 46 phosphoproteins and decreased the level of 23 phosphoproteins including 2 (Rac guanine nucleotide exchange factor T and SEC7 domain-containing protein) downregulated by more than 2-fold (Figure 3D–F; Supplementary File S4, Tabs 2–6). Phosphoproteins with missing values in one or more of the replicates were excluded from fold-change and statistical calculations but annotated and mentioned in the supplementary file separately (Supplementary File S4, Tab 7). For 5 phosphoproteins, both AprA and polyP decreased levels (Figure 2B; Supplementary File S7, Tab 7). One phosphoprotein (WW domain-containing protein), showed levels increased by both.

### 3.2. GO Term Analysis Reveals Overlapping and Distinct Pathways Regulated by AprA and polyP

To explore pathways regulated by AprA and polyP, we performed Gene Ontology (GO) and SMART domain enrichment analyses using all significantly upregulated and downregulated proteins and phosphoproteins. All enrichment results are provided in Supplementary File S5 (AprA) and File S6 (polyP), including top enriched pathways, GO terms for biological processes, molecular functions, and cellular components, as well as associated SMART domains. All accession numbers for significant proteins, including both characterized and uncharacterized entries, were submitted to ShinyGO for analysis.

To assess early signaling responses to AprA, we performed GO enrichment analyses for all significant proteins at 10 and 30 min. At 10 min, no GO terms passed the FDR threshold for enrichment, indicating that early responses were distributed across diverse functional categories. By 30 min, pathway-level organization began to emerge (Supplementary Figure S2A–D; Supplementary File S5, Tabs 2–6). Enriched biological process terms included peptide metabolic process, cellular amide metabolic process, and translation, while cellular component enrichment highlighted vesicle-associated categories, including intracellular, cytoplasmic, endocytic, and phagocytic vesicles. Molecular function enrichment was dominated by oxidoreductase activity, particularly aldehyde dehydrogenase (NAD^+^) activity. For polyP, no GO terms were significantly enriched at either 10 or 30 min, except for the 142 downregulated proteins at 10 min, which were enriched for proteins involved with ubiquitination and proteolysis (Supplementary File S6, Tab 2).

For 60 min AprA-regulated proteins, GO enrichment analysis revealed a dominant signature associated with RNA metabolism and ribosome biogenesis (Supplementary Figure S3A–C; Supplementary File S5, Tabs 7–10). Enriched biological processes included rRNA processing, small subunit of the ribosome (SSU)-rRNA maturation, ribosomal small-subunit biogenesis, mitochondrial RNA processing, and nucleolar RNA metabolism. Consistent with these functions, cellular component terms were highly enriched for nucleolus-associated ribosome assembly structures, including the 90S preribosome (a large precursor ribonucleoprotein complex that undergoes sequential processing to produce the small and large ribosomal subunits), small-subunit processome (a multiprotein complex required for early steps of SSU biogenesis), preribosome (a peripheral assembly intermediate associated with ribosome maturation) and ribonucleoprotein complexes, along with the nucleolus and nuclear lumen. GO molecular function analysis identified a single significant category, RNA binding (Supplementary Figure S3D; Supplementary File S5, Tab 11), suggesting an association between AprA-induced proteomic changes and transcriptional and translational control mechanisms.

PolyP-regulated proteins displayed a broadly similar enrichment profile, reflecting a shared impact on RNA-associated processes, but also exhibited distinctive features (Supplementary Figure S4A–D; Supplementary File S6, Tabs 3–7). Top biological processes included RNA surveillance and polyadenylation-dependent RNA catabolic pathways, as well as rRNA maturation and endonucleolytic cleavage events. Cellular component enrichment again highlighted ribosome assembly sites such as the preribosome, small-subunit processome, and nucleolus, alongside nuclear and membrane-enclosed lumens. Molecular function terms extended beyond RNA binding to include adenylyltransferase activity, suggesting that polyP signaling may influence RNA turnover and modification in addition to canonical ribosome biogenesis.

GO term analysis for the 31 proteins whose levels were affected by both AprA and polyP at 60 min showed a strong representation of ribosome biogenesis and RNA processing pathways, including endonucleolytic cleavage of rRNA, SSU-rRNA maturation, and ribosomal small-subunit biogenesis (Supplementary Figure S5A,B; Supplementary File S7, Tabs 3 and 4). Cellular component categories included nucleolar and ribosomal complexes (Supplementary Figure S5C; Supplementary File S7, Tab 5), while SMART domain enrichment identified ribosomal protein S1-like RNA-binding and helicase-associated domains (Supplementary Figure S5D; Supplementary File S7, Tab 6). Together, these results indicate that although AprA and polyP have largely distinct proteomic signatures, they converge on a functionally coherent group of proteins linked to ribosome assembly and RNA processing.

GO term analysis of the phosphoproteins indicated that at 10 min after AprA stimulation, phosphoproteomic enrichment was limited. GO molecular function analysis showed modest enrichment of RNA binding, GTPase regulator activity, nucleoside-triphosphatase regulator activity, and guanyl-nucleotide exchange factor activity, consistent with early modulation of signaling regulators. SMART domain analysis identified enrichment of the Sec7 domain, suggesting early regulation of Arf-family GEFs, and no biological process terms passed the FDR threshold at this time point (Supplementary Figure S6A–C; Supplementary File S5, Tabs 12–15).

By 30 min, AprA induced a broader and more coordinated phosphorylation signature. Enrichment expanded to include pathways and functions associated with Rho/Rac GTPase signaling (e.g., Rho/Rac cycles and GTPase regulation), along with translation initiation and cap-binding–related terms. SMART domain enrichment at 30 min included Sec7, PH-like, and additional signaling-linked domains (e.g., leucine-rich repeats and GTPase related regulatory motifs), consistent with increasing engagement of membrane trafficking, polarity and signaling modules as chemorepulsion becomes detectable (Supplementary Figure S7A–D; Supplementary File S5, Tabs 16–20).

For polyP, phosphoproteomic GO enrichment at 10 min did not yield significant pathway-level enrichment when all regulated phosphoproteins were considered together. However, stratified analysis revealed that upregulated phosphoproteins were selectively enriched for GTPase-related functions, whereas downregulated phosphoproteins were enriched for multiple pathways, including lipid- and RNA-associated processes (Supplementary File S6, Tab 8). At 30 min, enrichment remained limited but highlighted Sec7/ArfGEF-associated regulation, including Sec7 domain enrichment and ARF signaling–related terms, as well as domains linked to ubiquitin-associated and ERAD-related protein handling. This pattern suggests a more restricted and delayed phosphoregulatory response to polyP compared with AprA (Supplementary Figure S8A,B; Supplementary File S6, Tabs 9–11).

For AprA, enrichment analysis of the 83 phosphoproteins with significantly altered levels at 60 min (Supplementary Figure S9A; Supplementary File S5, Tab 22) highlighted pathways associated with ARF-protein signal transduction, cell cortex and leading-edge structures, FHA-containing proteins, and small-GTPase regulation. GO molecular functions (Supplementary Figure S9B; Supplementary File S5, Tab 23) emphasized guanyl-nucleotide exchange factor activity, GTPase regulator activity, mRNA binding, and enzyme regulator activity, consistent with coordinated control of GTPase cycling, actin-associated signaling, and transcript-linked regulation. SMART domain enrichment (Supplementary Figure S9C; Supplementary File S5, Tab 24) identified Sec7, PH, FHA, leucine-rich repeat, zinc-finger, ring-finger, and calponin homology (CH) domains, structural modules known to couple phosphoinositide-rich membranes to actin remodeling [49,50]. Together, these patterns suggest that AprA modulates phosphoproteins governing cortical architecture, phospholipid sensing, and small-GTPase–mediated cytoskeletal dynamics to promote chemorepulsion [51,52].

For polyP, enrichment of the 69 significantly altered phosphoproteins at 60 min (Supplementary Figure S10A; Supplementary File S6, Tab 13) highlighted regulators of intracellular trafficking and ARF/Rho-family GTPase signaling. GO molecular functions (Supplementary Figure S10B; Supplementary File S6, Tab 14) showed strong enrichment for phosphatidylinositol and phospholipid binding, guanyl-nucleotide exchange factor activity, and GTPase regulator activity, indicating effects on membrane–lipid interactions and GTPase cycling. Biological process terms (Supplementary Figure S10C; Supplementary File S6, Tab 15) emphasized regulation of ARF-mediated signal transduction, and SMART domains (Supplementary Figure S10D; Supplementary File S6, Tab 16) included Sec7, zinc-finger, and DEAD-like helicase domains, consistent with modulation of ArfGEF catalytic cores, metal-binding regulators, and RNA- or ATP-dependent helicase functions. These findings suggest that polyP influences phosphoproteins controlling membrane trafficking and small-GTPase–dependent signaling, key pathways driven by ARF and Rho GTPases [51,52].

**Figure 4:**
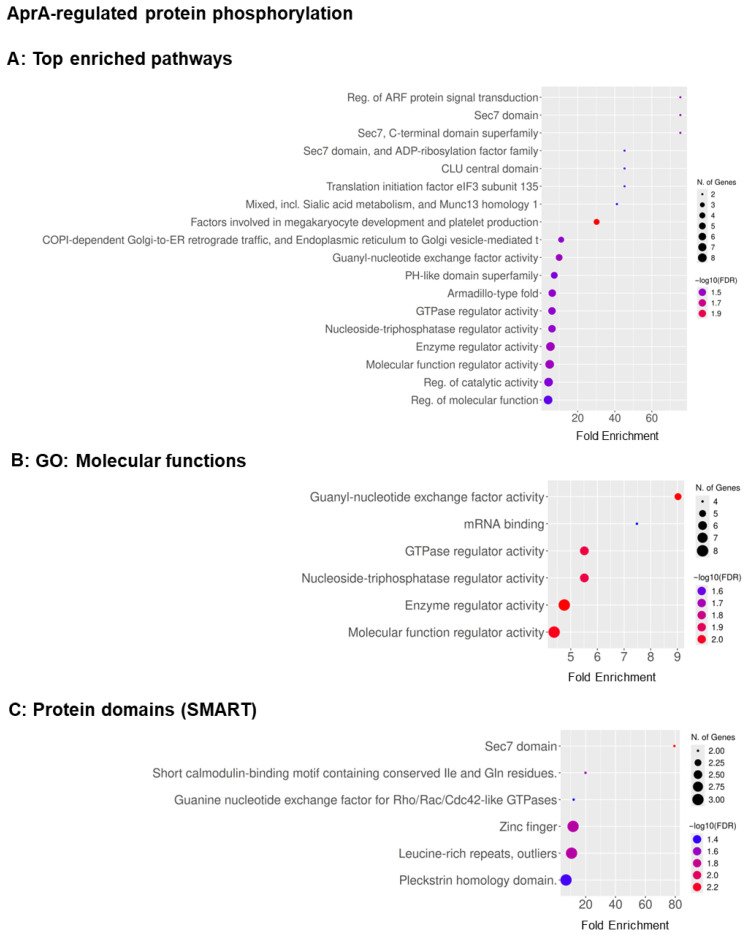
Functional enrichment of proteins whose phosphorylation is regulated by AprA at 60 min. (**A**) Top enriched pathways, (**B**) GO: Molecular Function, and (**C**) SMART protein domains. Bubbles show fold enrichment (*x*-axis); bubble size represents the number of genes; bubble color indicates statistical significance as −log10(FDR), ranging from blue (minimum) to red (maximum). FDR cutoff = 0.05, pathway size = 5–1000.

**Figure 5:**
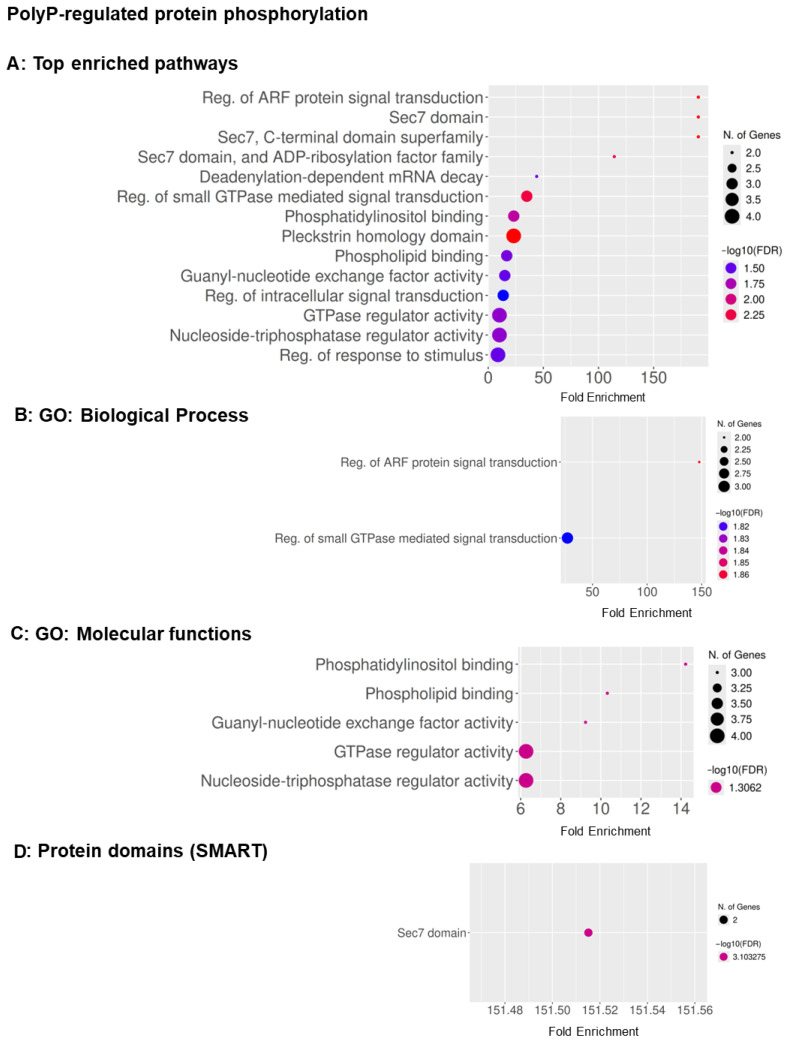
Functional enrichment of proteins whose phosphorylation is regulated by polyP at 60 min. (**A**) Top enriched pathways, (**B**) GO: Biological Process, (**C**) GO: Molecular Function, and (**D**) SMART protein domains. Bubbles show fold enrichment (*x*-axis); bubble size represents the number of genes; bubble color indicates statistical significance as −log10(FDR), ranging from blue (minimum) to red (maximum). FDR cutoff = 0.05, pathway size = 5–1000.

We next examined phosphoproteins whose phosphorylation at 60 min changed significantly relative to the change in levels of the protein. For AprA (51 proteins; Figure 4A–C and Supplementary File S5, Tabs 25–28), enriched categories included regulation of ARF-protein signal transduction, Sec7/Sec7-superfamily domains, guanyl-nucleotide exchange factor activity, GTPase regulator activity, and additional molecular-function terms related to enzyme binding and RNA-associated regulation. SMART-domain analysis further highlighted Sec7, PH-like, and pleckstrin-homology motifs, consistent with phospho-regulation of cortical and leading-edge ARF/Rac-GEFs that organize actin-rich regions required for chemorepulsion. For polyP (24 proteins; Figure 5A–D and Supplementary File S6, Tabs 17–21), enrichment similarly involved ARF and small-GTPase–mediated signal transduction but additionally extended to mRNA decay and RNA-processing pathways, phosphatidylinositol/phospholipid-binding functions, and guanyl-nucleotide exchange factor activity. SMART-domain analysis was dominated by the Sec7 catalytic core, suggesting links between polyP signaling, signal transduction, and post-transcriptional regulation.

The phosphorylation of 6 proteins was affected by both AprA and polyP at 60 min (Figure 2B; Supplementary File S7, Tab 7). These included Sec7-domain (ArfGEF) proteins, the Ras-interacting protein RipA (UniProtKB #C7G030), the RacGEF GxcT (UniProtKB # Q55DL8), a WW domain-containing protein, which is typically involved in organizing multiprotein complexes important for signaling and organelle dynamics [53], and a Clu-domain–containing protein, which is typically involved in organizing multiprotein complexes important for signaling and organelle dynamics [54]. Both AprA and polyP decreased the phosphorylation of five of these proteins, whereas the phosphorylation of WW domain-containing protein was increased by both AprA and polyP. When phosphorylation sites were examined, AprA regulated 129 significant phosphorylation sites, whereas polyP affected 105 such sites (Supplementary File S7, Tabs 9 and 10). Among these, 8 sites were common among the overlapping phosphoproteins from both treatments. Collectively, these overlapping proteins and their eight shared phosphorylation sites (Supplementary File S7, Tabs 7 and 8) map to regulators of small GTPase signaling, membrane trafficking, and cytoskeletal remodeling, all core processes in directed cell movement [51,52,55]. Together, the results indicate potential points of signaling crosstalk and bifurcation between the AprA and polyP chemorepulsion pathways.

### 3.3. Two Proteins, GxcT and RipA, with Repellent-Decreased Phosphorylation Are Necessary for Chemorepulsion

**Figure 6:**
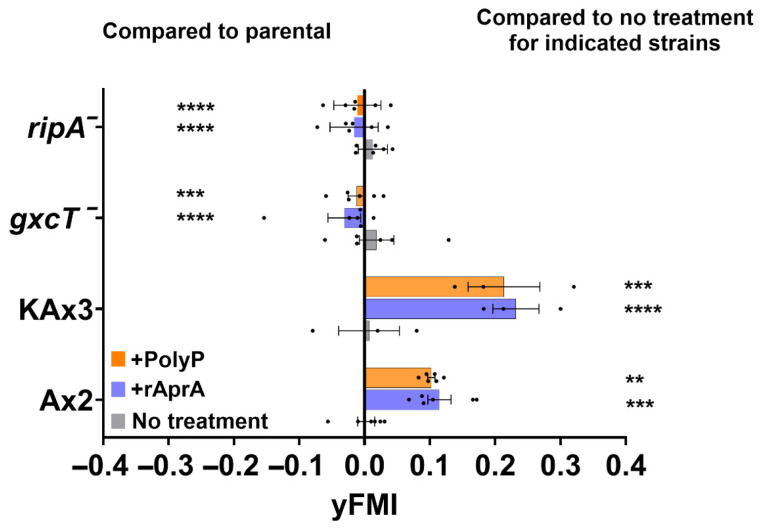
Chemotactic responses of phosphoprotein mutants in AprA and polyP gradients. Forward Migration Index (yFMI) along the gradient axis for parental (Ax2 and KAx3) and phosphoprotein knockout strains (*gxcT*^−^ and *ripA*^−^) respectively. Cells were exposed to AprA (blue), polyP (orange), or HL5 media as no treatment (gray). Positive yFMI values indicate repulsion from the source, while negative values indicate attraction. Bars are mean ± SEM, *n* ≥ 3. ** *p* < 0.01; *** *p* < 0.001; **** *p* < 0.0001 (Two-way ANOVA with Tukey’s multiple comparisons test among three different groups within respective parental and mutant, analyzed relative to parental (left column) and to untreated controls within each strain (right column).

To assess the functional relevance of candidate phosphoregulated proteins identified in our phosphoproteomic analysis, we performed chemotaxis assays using strains with targeted knockouts of two candidate genes hypophosphorylated at 60 min after AprA or polyP exposure: *gxcT* (a Rac GEF; [31], and *ripA* (a Ras-interacting protein and TORC2 component; [30]. The primary metric was the forward migration index (FMI), which is the net displacement of cells along the gradient axis divided by the total path length [56]. Here, negative FMI indicates attraction toward the source, while positive FMI indicates repulsion. As previously observed [10,12] parental Ax2 (the parental strain for *gxcT*^−^) and KAx3 (the parental strain for *ripA*^−^) were repelled by AprA and polyP (Figure 6). In both AprA and polyP gradients, *gxcT*^−^ and *ripA*^−^ cells showed no significant chemorepulsion (Figure 6). To determine if the lack of chemorepulsion of *gxcT*^−^ and *ripA*^−^ cells was due to a general defect in cell motility, we measured migration speed and directionality (Euclidean distance divided by total distance) (Supplementary Figure S11). Although the mutants showed slight differences from parental cells, they still showed motility, suggesting that the chemorepulsion defect in *gxcT*^−^ and *ripA*^−^ cells is not due to a general impairment of motility.

### 3.4. GxcT and RipA Do Not Mediate AprA and polyP Proliferation Inhibition

**Figure 7:**
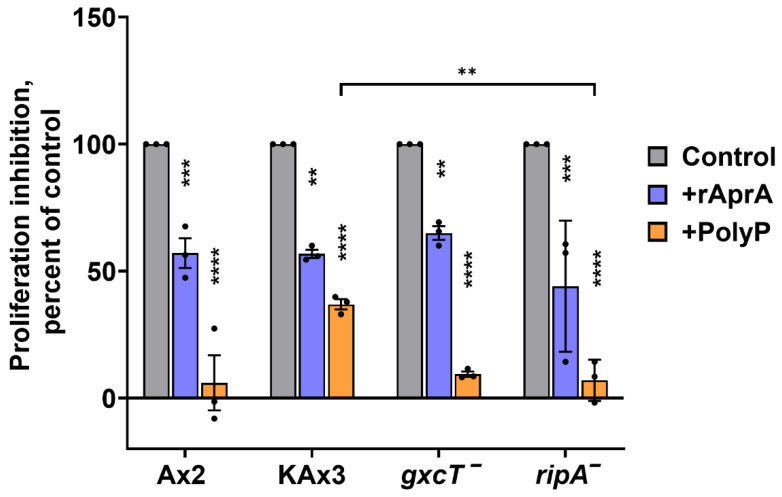
Proliferation inhibition analysis of wild-type and mutant *D. discoideum* strains. Proliferation inhibition was quantified for the indicated strains after 24 h in HL5 medium with AprA (blue), polyP (orange), or untreated control (gray). Values are the percentage of untreated controls. Bars are mean ± SEM, *n* = 3. ** *p* < 0.01; *** *p* < 0.001; **** *p* < 0.0001 (Two-way ANOVA with Sidak’s multiple comparisons test among three different groups within respective parental and mutant).

In addition to inducing repulsion, AprA and polyP inhibit *D. discoideum* cell proliferation. As previously observed [16,20], 300 ng/mL AprA and 700 µg/mL polyP reduced proliferation of Ax2 cells (Figure 7). Both the mutant strains showed proliferation inhibition by AprA and polyP (although for unknown reasons *ripA*^−^ was more sensitive to polyP than its parental KAx3), indicating that GxcT and RipA are part of the chemorepulsion mechanism and do not significantly mediate proliferation inhibition.

## 4. Discussion

Possibly as a way to deal with an extracellular environment where a signal might interact with the substrate and not diffuse properly, *D. discoideum* cells use two different chemorepellent signals to cause dispersal of cells from a growing colony. We previously found, by examining cell behavior and mutants lacking known signal transduction pathway components, that the two repellents use a combination of unique and overlapping pathway components to activate different chemorepulsion mechanisms. In this report, using an unbiased proteomics and phosphoproteomics approach, we find additional evidence supporting the differences in how AprA and polyP affect cells. In addition to the differences, there were some similarities, as the levels of a relatively small number of proteins, and the phosphorylation of a relatively small number of proteins, were regulated by both repellents. As with any proteomics or phosphoproteomics study, the quantitated proteins are only the most abundant proteins, and there are probably many more proteins whose levels and/or phosphorylation are regulated by AprA and/or polyP. Another important caveat in this study is that the cells were exposed to a constant level of the repellents, rather than a gradient, since there is currently no physical way to expose large numbers of cells (needed for proteomics and phosphoproteomics) to the same extracellular gradient. There may thus be some different effects when cells are exposed to a spatial gradient rather than a constant level of the repellent. However, two of the proteins where both repellents decreased phosphorylation were necessary for repulsion by both repellents, indicating that for at least some proteins, a repellent-induced change in phosphorylation is associated with a functional role in repulsion.

At the edge of a colony, cells will be in a concentration of AprA or polyP that increases slowly as the colony size increases, but as these cells move away from the colony, the AprA or polyP concentration will decrease. Thus, unlike the pulsatile attractant cAMP, whose concentration at a given cell rises and falls within a few minutes [57,58], the repellent concentrations change relatively slowly over time. Proteomic and phosphoproteomic changes were detectable at 10 and 30 min, indicating early signaling responses to chemorepellents. By 60 min, a larger and more stable set of regulated proteins and phosphorylation events was observed, likely reflecting a combination of early signaling and downstream adaptive responses, which enabled more robust pathway-level interpretation.

After 60 min, we observed 51 proteins whose phosphorylation was affected by AprA and 24 proteins whose phosphorylation was affected by polyP. Although this is considerably less than the almost 700 *D. discoideum* proteins whose phosphorylation was significantly altered by the chemoattractant cAMP [59], it indicates that the chemorepellents affect a considerable number of proteins. For the proteomics, one unexpected result was that both repellents downregulated RNA metabolism proteins and upregulated ribosomal biogenesis proteins. This suggests that high levels of the two extracellular signals, indicating the presence of a large number of nearby cells, causes a cell to prepare to change its proteome from the proteome it had when it was a relatively isolated cell, perhaps derived from a dispersed spore. Both repellents affected, as expected, phosphorylation of signal transduction pathway components and unexpectedly affected phosphorylation of RNA processing proteins, albeit generally different sets of proteins. Combined with the effects on levels of ribosomal biogenesis proteins, the effects on phosphorylation of RNA processing proteins indicate that the two signals affect mRNA translation.

The phosphoproteomics profiles suggest that AprA and polyP influence distinct but partially overlapping signaling processes that could induce chemorepulsion. Both repellents reduced phosphorylation of proteins associated with Arf GTPases and Sec7-domain–containing guanine nucleotide exchange factors (ArfGEFs), which regulate vesicle trafficking, membrane remodeling, and actin cytoskeleton dynamics processes essential for directed cell migration [51,60]. Despite this shared regulation, AprA inhibits pseudopod formation at the side of the cell closest to the source of AprA whereas polyP promotes pseudopod extension at the side furthest from the source of polyP [4,10,12]. One apparent difference is that PolyP but not AprA affected phosphorylation of phosphatidylinositol binding proteins, which is consistent with the observation that polyP but not AprA requires phospholipase C and PI3 kinase for repulsion [12].

Both *gxcT*^−^ and *ripA*^−^ cells failed to migrate away from AprA or polyP, indicating that these two proteins are required for chemorepulsion in response to both signals. GxcT encodes a Rho-family guanine-nucleotide exchange factor that stabilizes directional Ras activation and PIP_3_ production during chemotaxis independently of the actin cytoskeleton [31]. Loss of GxcT likely disrupts Rac-mediated cytoskeletal organization and polarity maintenance necessary for effective migration away from repellent cues. RipA (also known as Rip3/SIN1) acts as a Ras-interacting scaffold within the TORC2 complex, where it regulates F-actin dynamics and cell polarity [30,61,62]. The loss of repulsion in *ripA*^−^ cells suggests defective TORC2-mediated cytoskeletal control downstream of Ras signaling. Together, these findings suggest that AprA and polyP converge on shared Ras- and Rac-GTPase modules through RipA- and GxcT-dependent regulation of TORC2 and cytoskeletal polarity, in addition to other signal transduction pathway components needed by both repellents [12]. Since the chemorepulsion of human neutrophils uses several of the signal transduction pathway components used by AprA [63], there is an intriguing possibility that human neutrophil chemorepulsion also involves some of the proteomics changes and phosphorylation changes identified in this report.

Although specific antibodies for RipA and GxcT are not currently available, preventing direct biochemical validation of individual protein or phosphorylation sites identified in this study, the requirement for both proteins in AprA- and polyP-induced chemorepulsion is supported by the loss-of-function phenotypes observed in the corresponding knockout strains. Notably, the regulated phosphorylation sites identified in RipA and GxcT occur within protein domains previously implicated in Ras- and TORC2-dependent signaling, suggesting potential functional relevance [30,31,61]. Future studies using phospho-null and phospho-mimetic mutants will be necessary to determine whether specific phosphorylation events directly mediate chemorepulsion. Notably, many of the conserved signaling modules implicated here, including Ras/Rac GTPase regulation, ArfGEF-dependent trafficking, and TORC2-mediated polarity control, also play central roles in directional migration and invasion of cancer cells, underscoring the value of *Dictyostelium* as a model for fundamental eukaryotic motility pathways.

## Data Availability

Raw and processed proteomic and phosphoproteomic datasets have been deposited in the MassIVE repository at the University of California, San Diego Center for Computational Mass Spectrometry under accession number MSV000098894 (https://massive.ucsd.edu/ProteoSAFe/private-dataset.jsp?task=e3e85c8f691040a3a5da1405b5acd97f, accessed on 20 August 2025). Supplementary data files associated with this study are available on Figshare at https://figshare.com/s/01204b40574a142e3037, accessed on 21 October 2025.

## Supplementary Materials

The following supporting information can be downloaded at: https://www.mdpi.com/article/10.3390/cells15010060/s1, Supplementary Figure S1: Determination of recombinant AprA concentration by SDS–PAGE densitometry; Supplementary Figure S2: Functional enrichment of proteins regulated by AprA at 30 min; Supplementary Figure S3: Functional enrichment of proteins regulated by AprA at 60 min; Supplementary Figure S4: Functional enrichment of proteins regulated by polyP at 60 min; Supplementary Figure S5: Functional enrichment of overlapping proteins regulated by both AprA and polyP at 60 min; Supplementary Figure S6: Functional enrichment of phosphoproteins regulated by AprA at 10 min; Supplementary Figure S7: Functional enrichment of phosphoproteins regulated by AprA at 30 min; Supplementary Figure S8: Functional enrichment of phosphoproteins regulated by polyP at 30 min; Supplementary Figure S9: Functional enrichment of AprA-regulated phosphoproteins at 60 min; Supplementary Figure S10: Functional enrichment of PolyP-regulated phosphoproteins at 60 min; Supplementary Figure S11: Chemotactic responses of phosphoprotein mutants in AprA and polyP gradients; Supplementary File S1: AprA total proteins; Supplementary File S2: PolyP total proteins; Supplementary File S3: AprA phosphoproteins; Supplementary File S4: PolyP phosphoproteins; Supplementary File S5: Enrichment analysis of AprA-regulated proteins and phosphoproteins; Supplementary File S6: Enrichment analysis of PolyP- regulated proteins and phosphoproteins; Supplementary File S7: Proteins, phosphoproteins, and phosphosites regulated by both AprA and PolyP.
