# Supplementary material for: Phosphoproteomic Profiling Reveals Overlapping and Distinct Signaling Pathways in Dictyostelium discoideum in Response to Two Different Chemorepellents
Source: Cells. 2025 Dec 29;15(1):60. doi: 10.3390/cells15010060 (PMC12785797; doi:10.3390/cells15010060)
Supplement: Supplementary file 1 [file cells-15-00060-s001.zip › Supplementary Figures.pdf]

Supplementary Figure S1: Determination of recombinant AprA concentration by SDS–PAGE densitometry

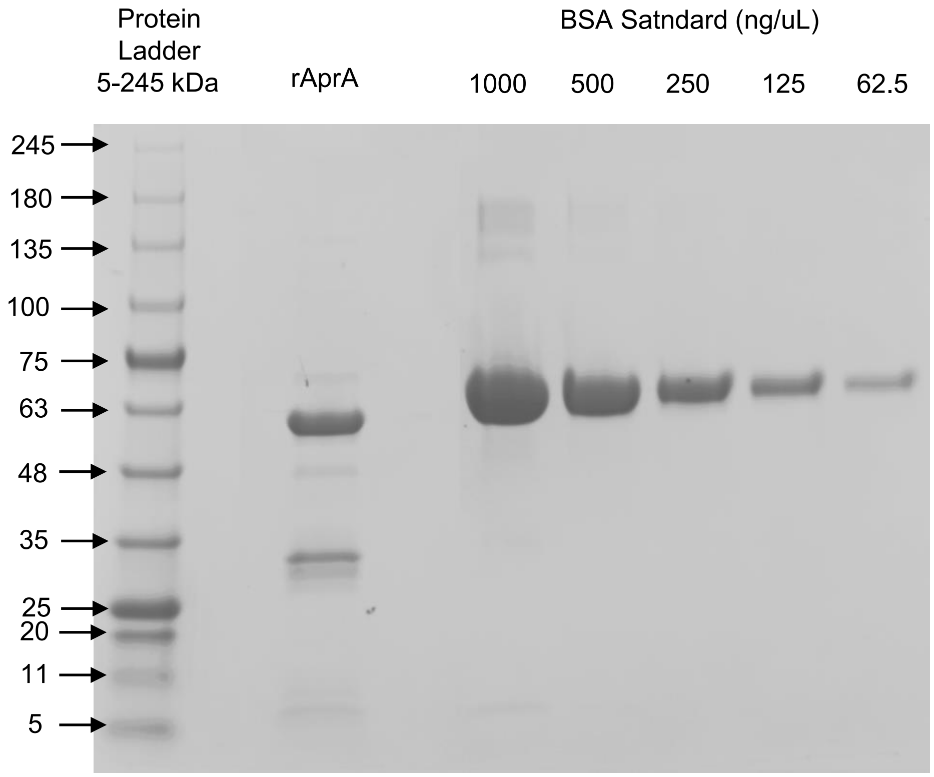

Supplementary Figure S1. Determination of recombinant AprA concentration by SDS–PAGE densitometry. Coomassie-stained SDS–PAGE gel showing purified recombinant AprA (rAprA) alongside a bovine serum albumin (BSA) standard curve. Known amounts of BSA (62.5–1000 ng) were loaded to generate a calibration series for densitometric quantification. The rAprA sample migrates primarily at ~55–60 kDa, consistent with the predicted molecular mass of AprA, with additional lower-molecular-weight bands likely representing processed or truncated forms. Band intensities were quantified by densitometry and compared to the BSA standard curve to estimate rAprA concentration for use in stimulation and chemorepulsion experiments.

Supplementary Figure S2: Functional enrichment of proteins regulated by AprA at 30 minutes

A: Top enriched pathways

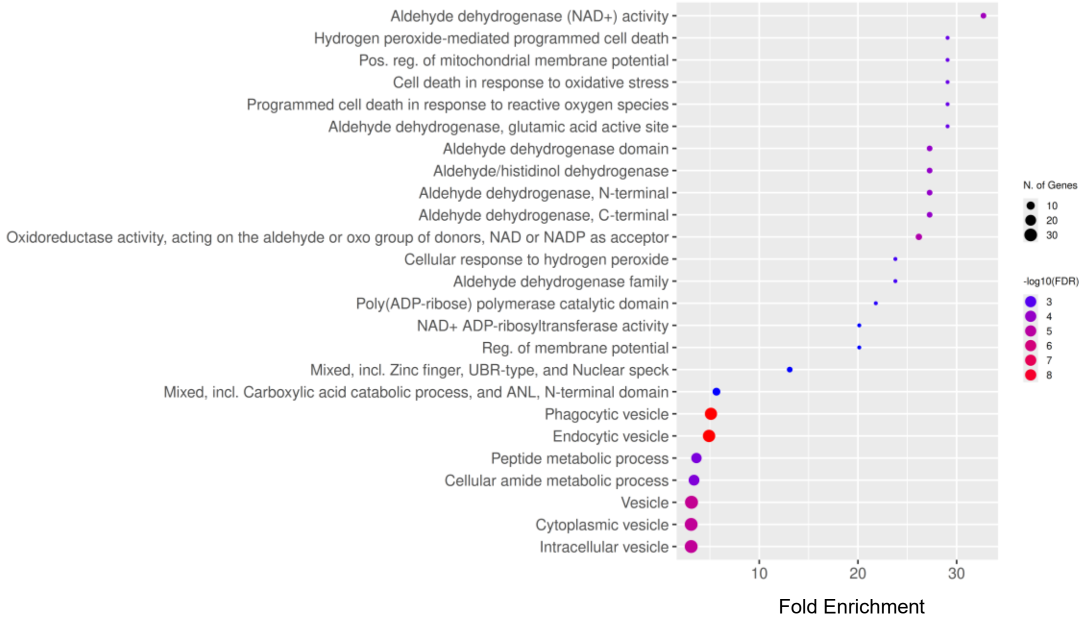

B: GO: Biological Process

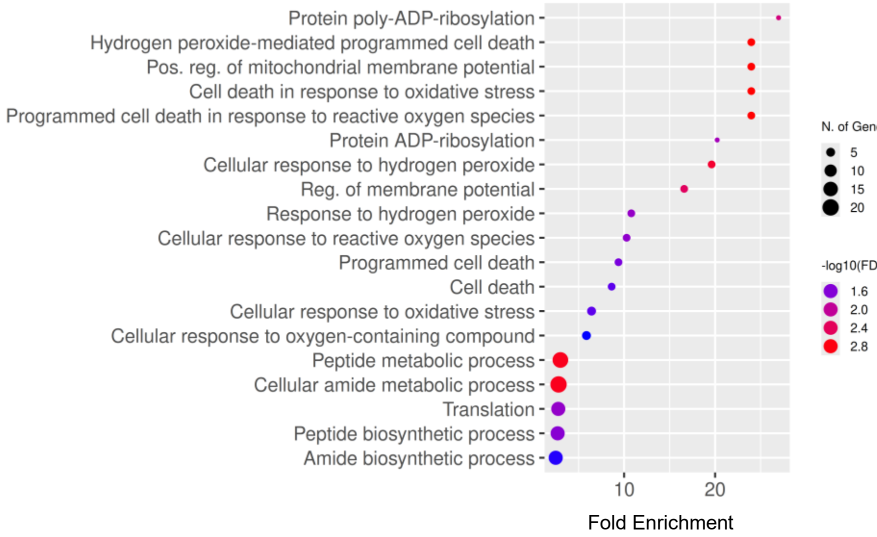

C: GO: Cellular Component

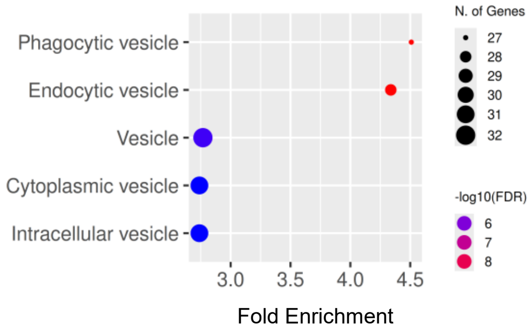

D: GO: Molecular function

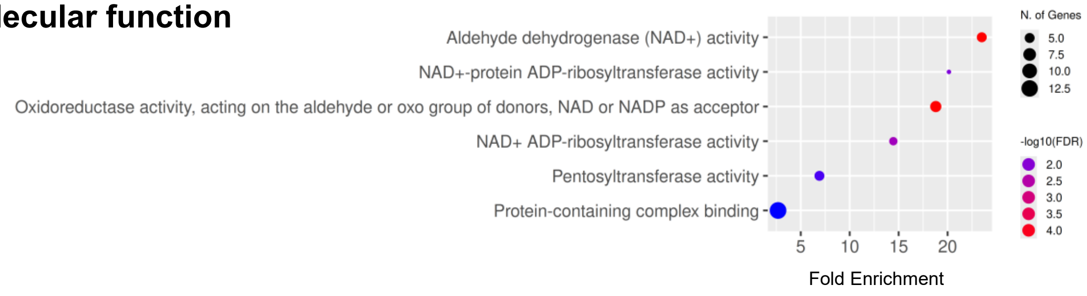

Supplementary Figure S2. Functional enrichment of proteins whose levels are regulated by AprA at 30 minutes. (**A**) Top enriched pathways, (**B**) GO: Biological Process, (**C**) GO: Cellular Component, and (**D**) GO: Molecular Function. Bubbles show fold enrichment (x-axis); bubble size represents the number of genes; bubble color indicates statistical significance as –log10(FDR), ranging from blue (minimum) to red (maximum). FDR cutoff = 0.05, pathway size = 5–1000.

Supplementary Figure S3: Functional enrichment of proteins regulated by AprA at 60 minutes

A: Top enriched pathways

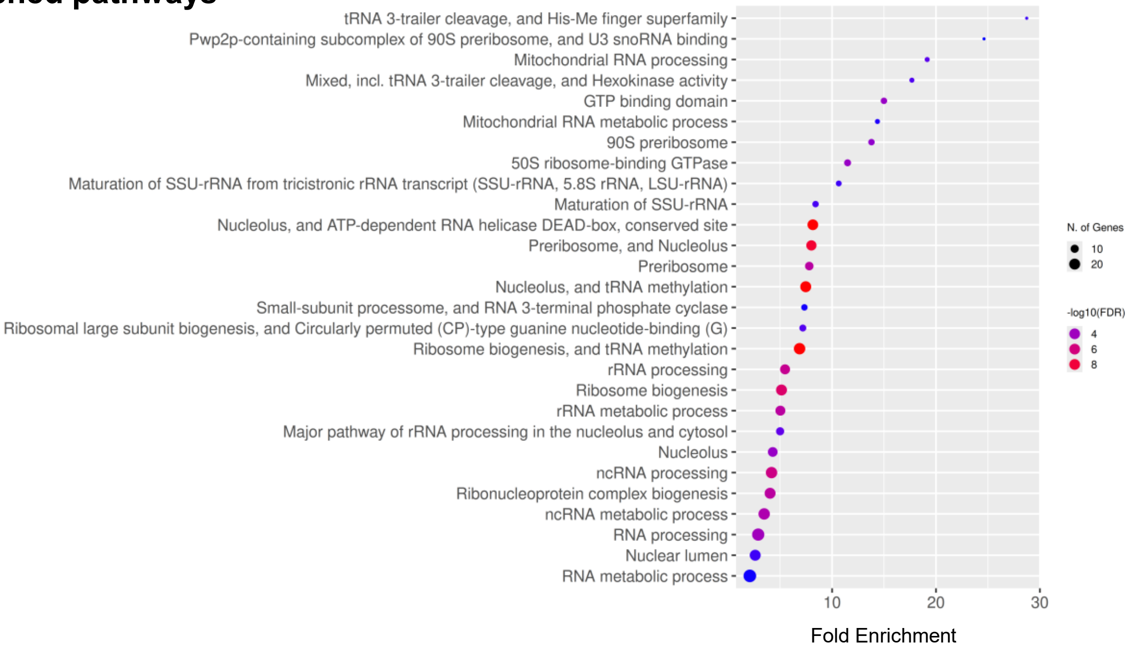

B: GO: Biological Process

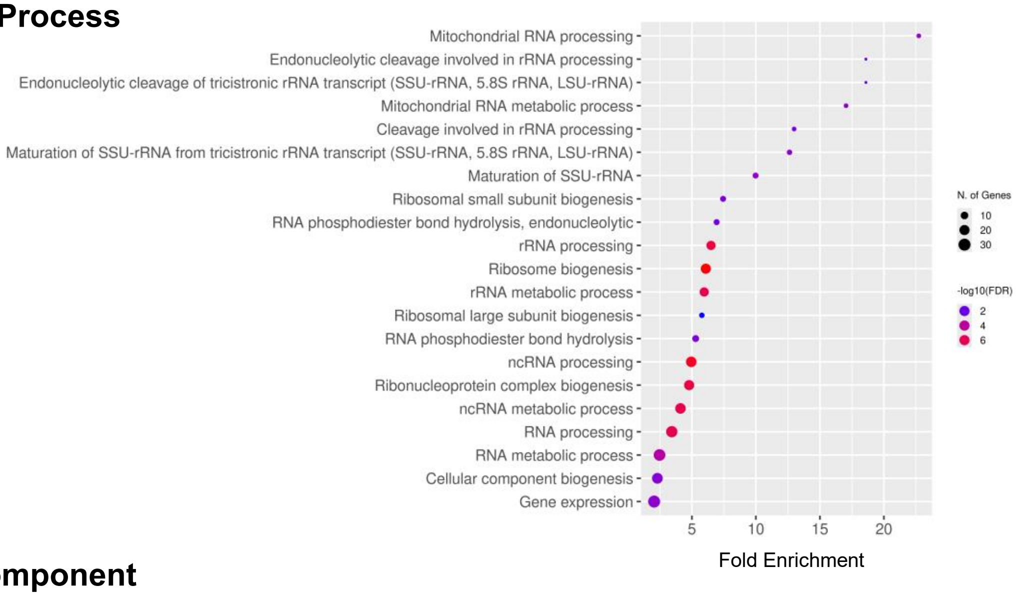

C: GO: Cellular Component

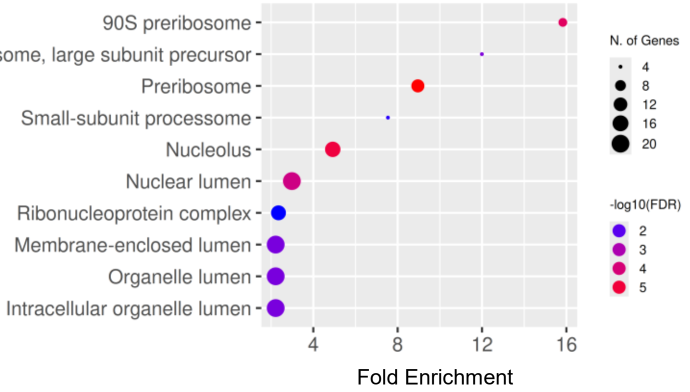

D: GO: Molecular function

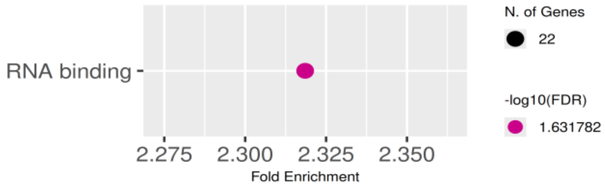

Supplementary Figure S3. Functional enrichment of proteins whose levels are regulated by AprA at 60 minutes. (**A**) Top enriched pathways, (**B**) GO: Biological Process, (**C**) GO: Cellular Component, and (**D**) GO: Molecular Function. Bubbles show fold enrichment (x-axis); bubble size represents the number of genes; bubble color indicates statistical significance as –log10(FDR), ranging from blue (minimum) to red (maximum). FDR cutoff = 0.05, pathway size = 5–1000.

Supplementary Figure S4: Functional enrichment of proteins regulated by polyP at 60 minutes

A: Top enriched pathways

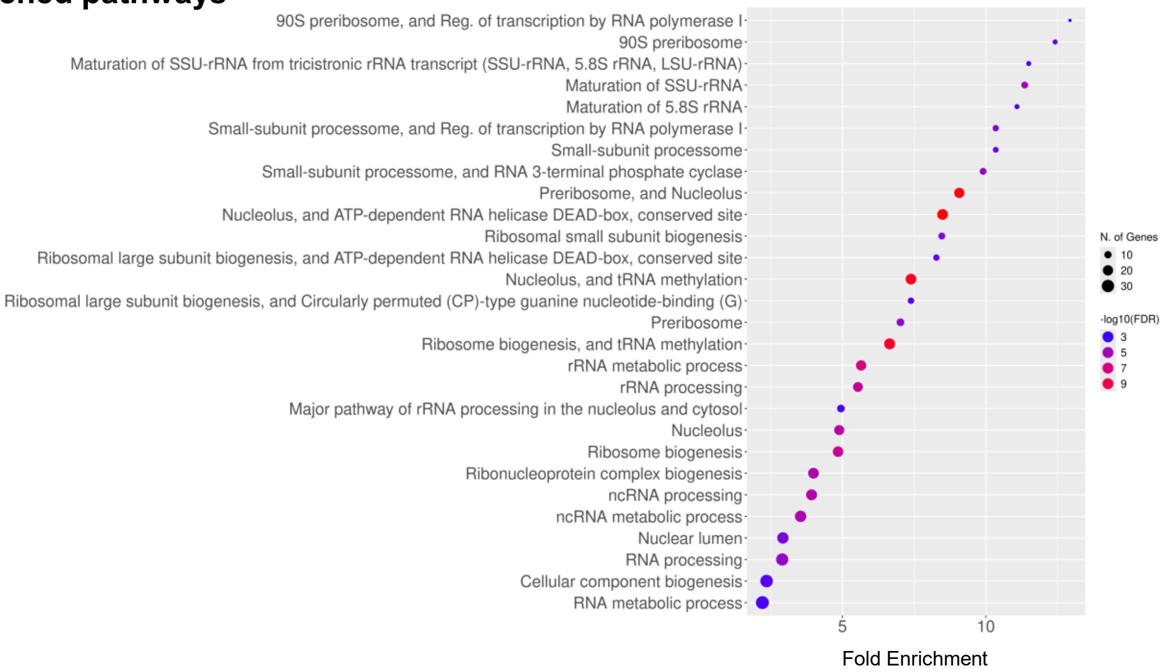

B: GO: Biological Process

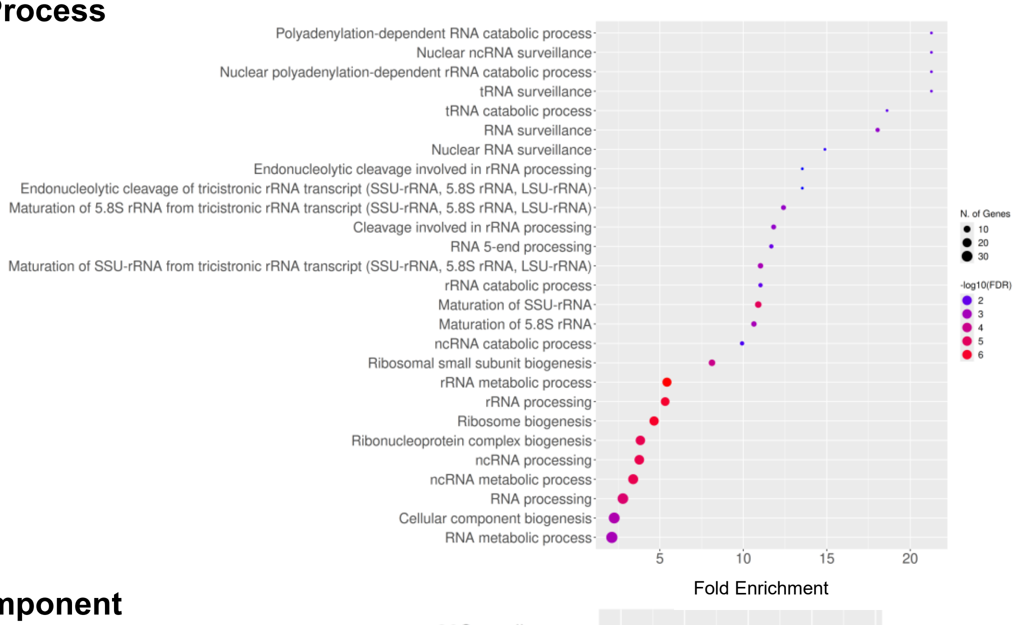

C: GO: Cellular Component

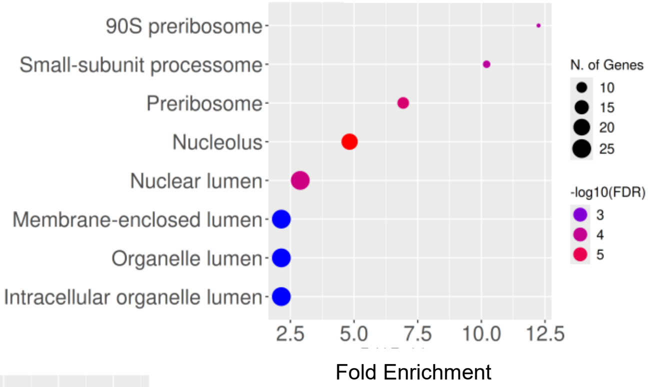

D: GO: Molecular function

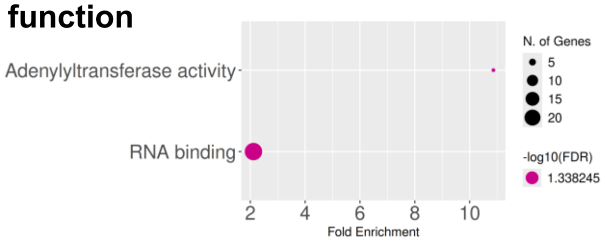

Supplementary Figure S4. Functional enrichment of proteins whose levels are regulated by polyp at 60 minutes. (**A**) Top enriched pathways, (**B**) GO: Biological Process, (**C**) GO: Cellular Component, and (**D**) GO: Molecular Function. Bubbles show fold enrichment (x-axis); bubble size represents the number of genes; bubble color indicates statistical significance as –log10(FDR), ranging from blue (minimum) to red (maximum). FDR cutoff = 0.05, pathway size = 5–1000.

Supplementary Figure S5: Functional enrichment of overlapping proteins regulated by both AprA and polyP at 60 minutes

A: Top enriched pathways

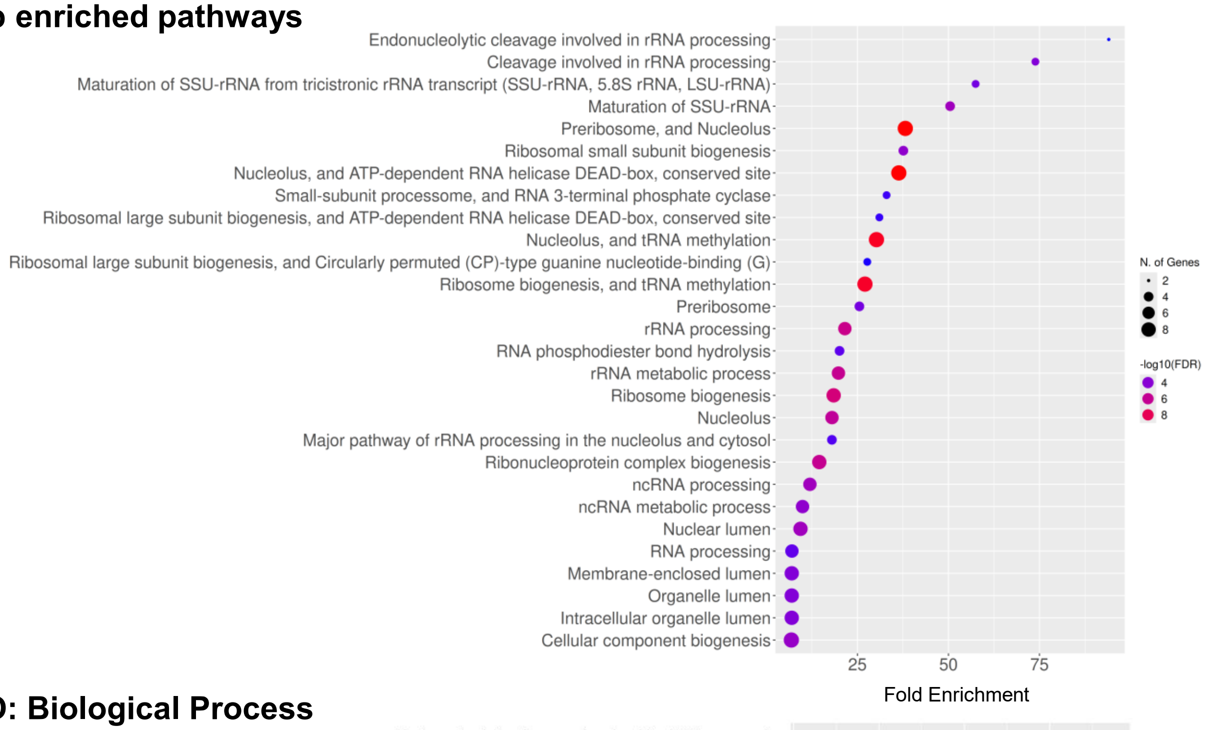

B: GO: Biological Process

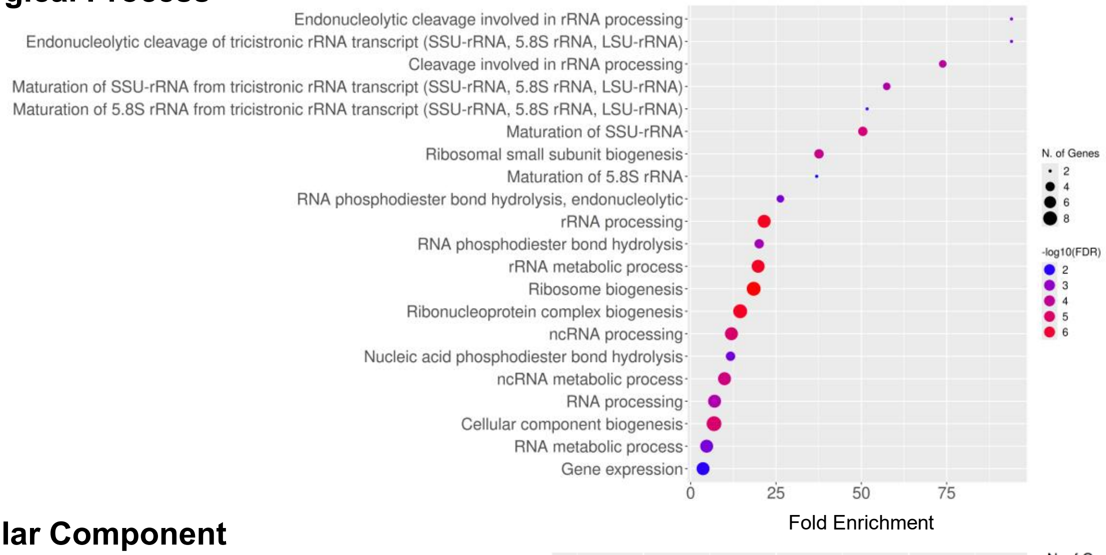

C: GO: Cellular Component

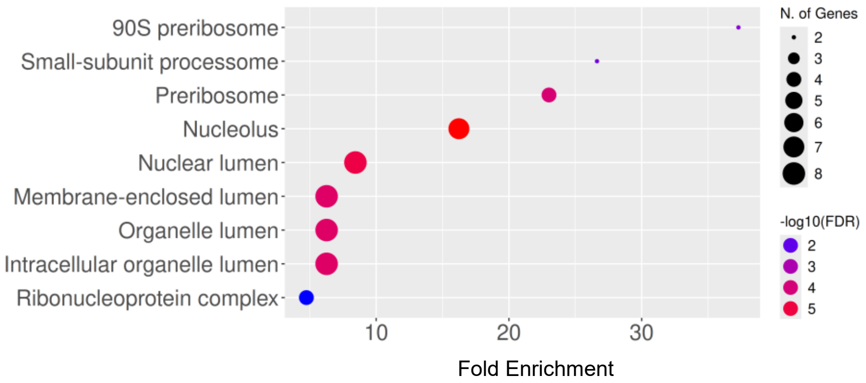

D: Protein domains (SMART)

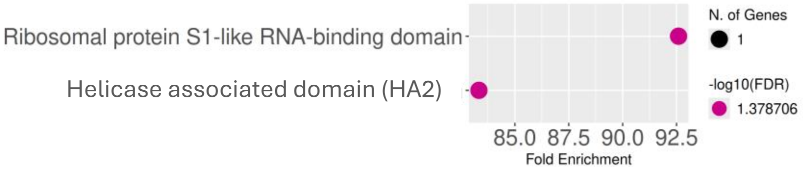

Supplementary Figure S5. Functional enrichment of proteins whose levels are regulated by both AprA and polyP at 60 minutes. (**A**) Top enriched pathways, (**B**) GO: Biological Process, (**C**) GO: Cellular Component, and (**D**) SMART protein domains. Bubbles show fold enrichment (x-axis); bubble size represents the number of genes; bubble color indicates statistical significance as –log10(FDR), ranging from blue (minimum) to red (maximum). FDR cutoff = 0.05, pathway size = 5–1000. No GO: Molecular Function was enriched for this group of proteins.

Supplementary Figure S6: Functional enrichment of phosphoproteins regulated by AprA at 10 minutes

A: Top enriched pathways

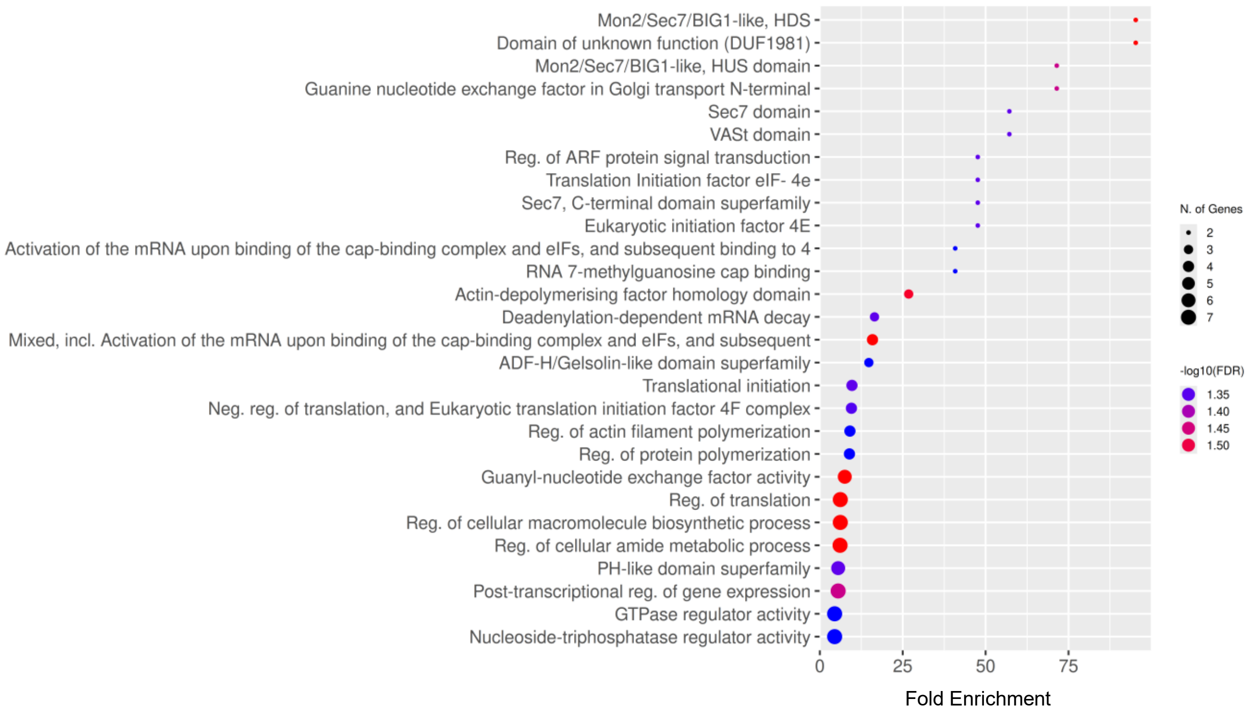

B: GO: Molecular functions

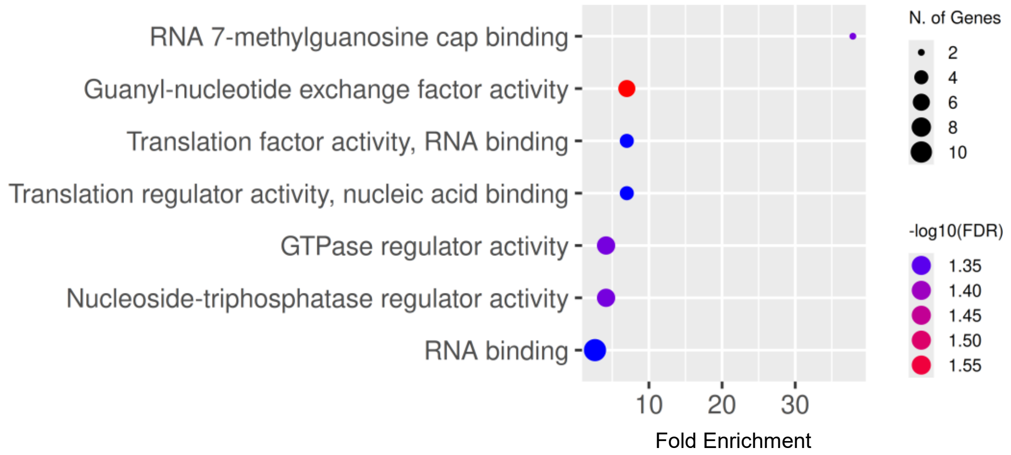

C: Protein domains (SMART)

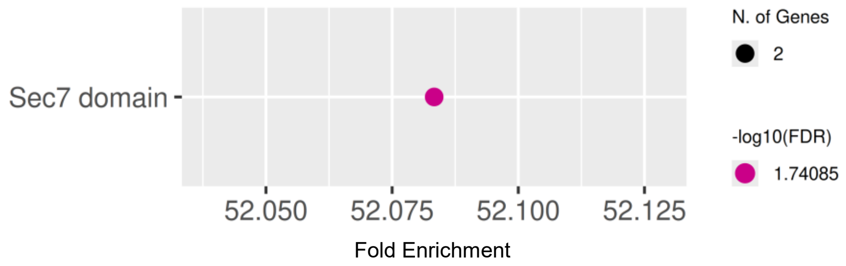

Supplementary Figure S6. Functional enrichment of phosphoproteins whose levels are regulated by AprA at 10 minutes. (**A**) Top enriched pathways, (**B**) GO: Molecular Function, and (**C**) SMART protein domains. Bubbles show fold enrichment (x-axis); bubble size represents the number of genes; bubble color indicates statistical significance as –log10(FDR), ranging from blue (minimum) to red (maximum). FDR cutoff = 0.05, pathway size = 2–1000.

Supplementary Figure S7: Functional enrichment of phosphoproteins regulated by AprA at 30 minutes

A: Top enriched pathways

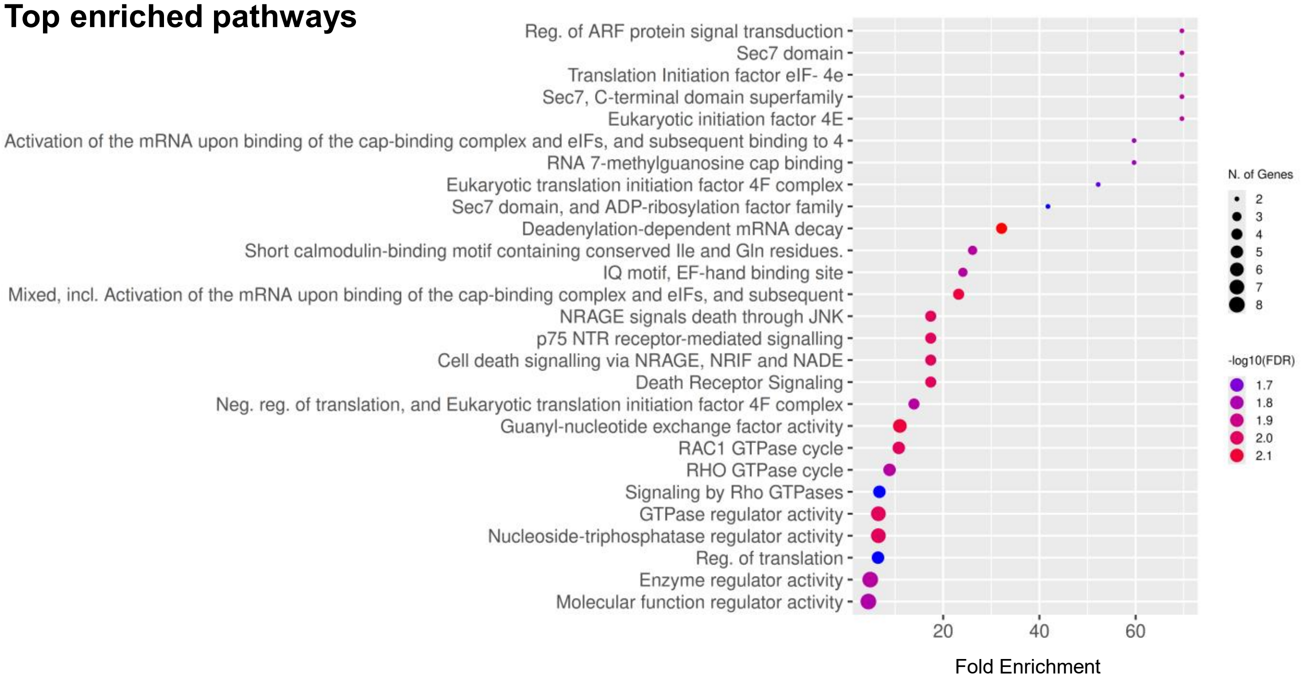

B: GO: Cellular component

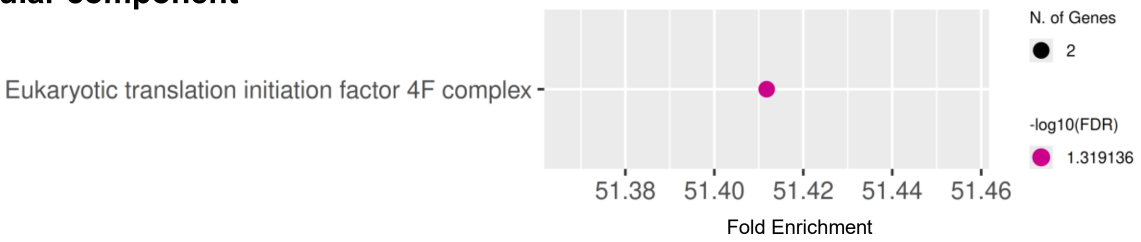

C: GO: Molecular functions

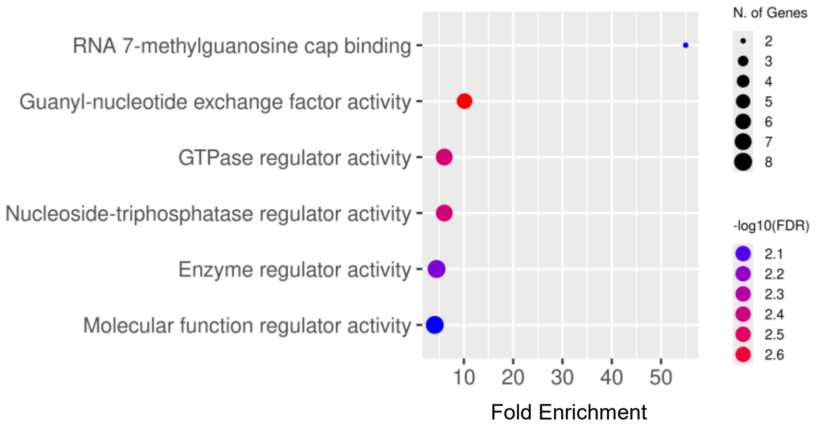

D: Protein domains (SMART)

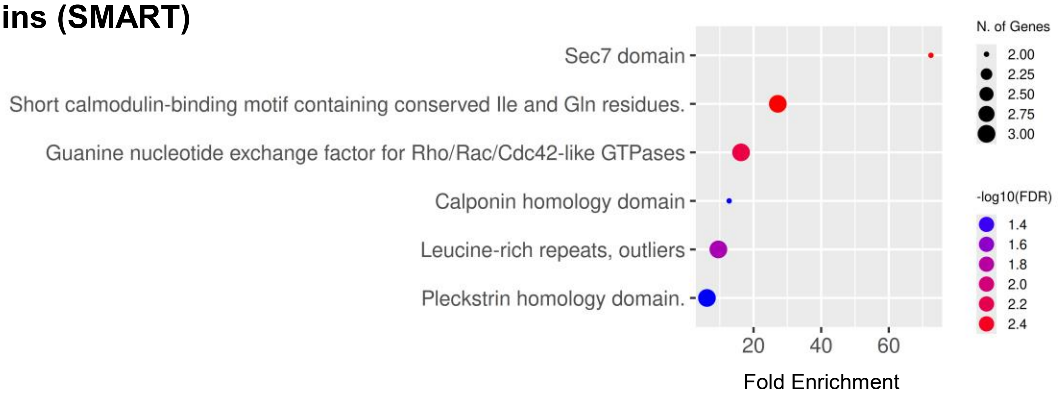

Supplementary Figure S7. Functional enrichment of phosphoproteins whose levels are regulated by AprA at 30 minutes. (**A**) Top enriched pathways, (**B**) GO: Cellular Component, (**C**) GO: Molecular Function, and (**D**) SMART protein domains. Bubbles show fold enrichment (x-axis); bubble size represents the number of genes; bubble color indicates statistical significance as –log10(FDR), ranging from blue (minimum) to red (maximum). FDR cutoff = 0.05, pathway size = 5–1000.

Supplementary Figure S8: Functional enrichment of phosphoproteins regulated by polyP at 30 minutes

A: Top enriched pathways

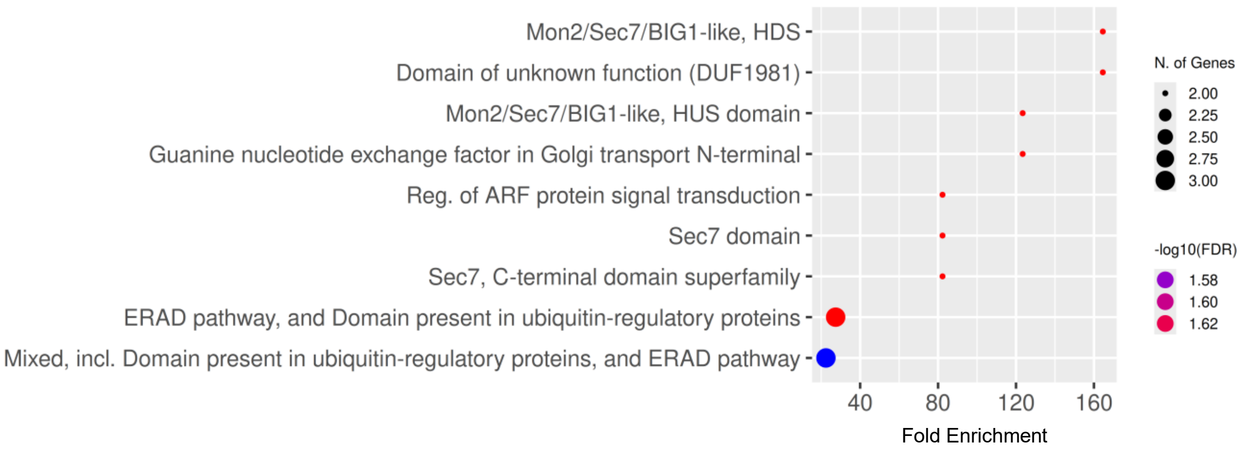

B: Protein domains (SMART)

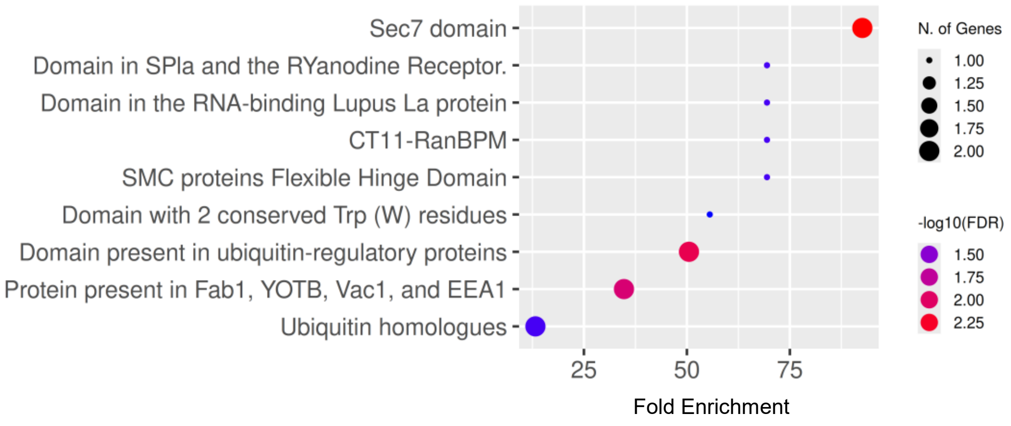

Supplementary Figure S8. Functional enrichment of phosphoproteins whose levels are regulated by polyP at 30 minutes. (**A**) Top enriched pathways, and (**B**) SMART protein domains. Bubbles show fold enrichment (x-axis); bubble size represents the number of genes; bubble color indicates statistical significance as –log10(FDR), ranging from blue (minimum) to red (maximum). FDR cutoff = 0.05, pathway size = 5–1000.

Supplementary Figure S9: Functional enrichment of AprA-regulated phosphoproteins at 60 minutes

A: Top enriched pathways

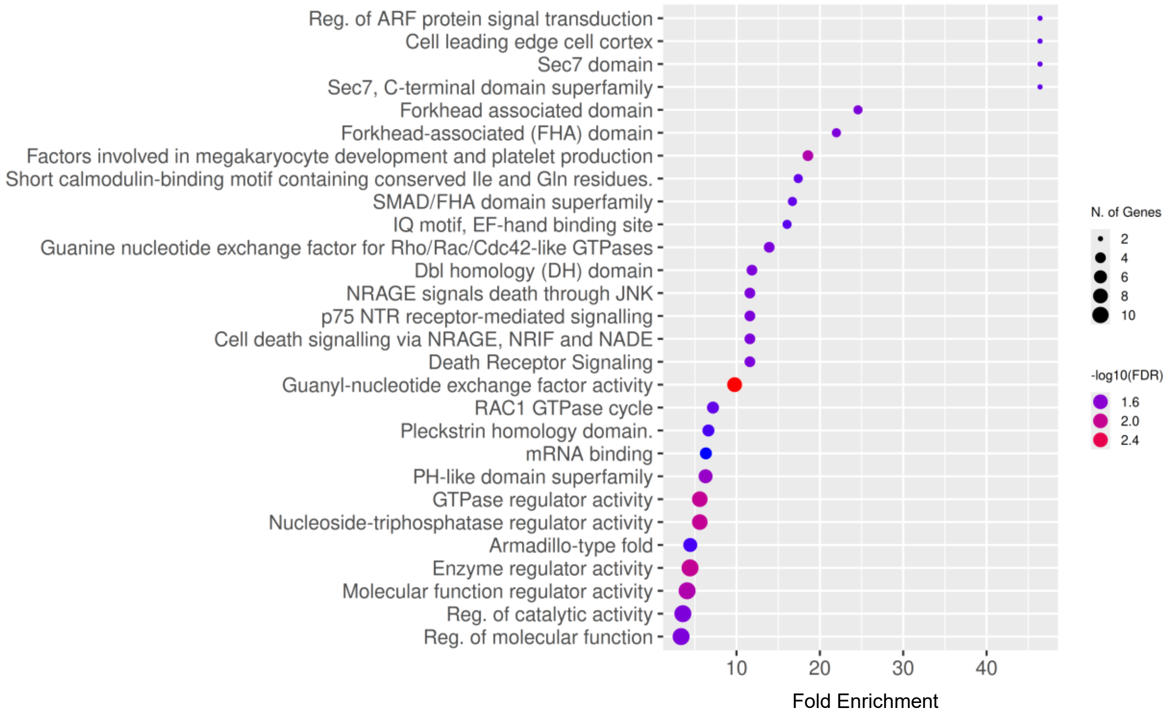

B: GO: Molecular functions

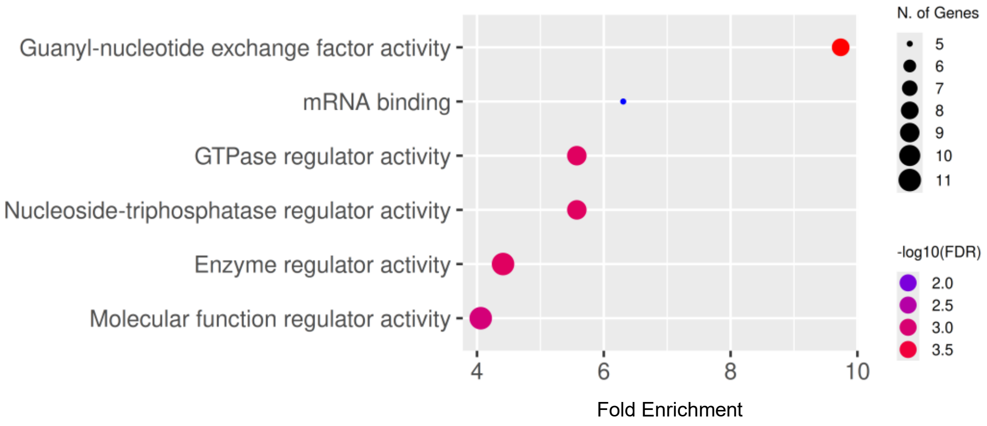

C: Protein domains (SMART)

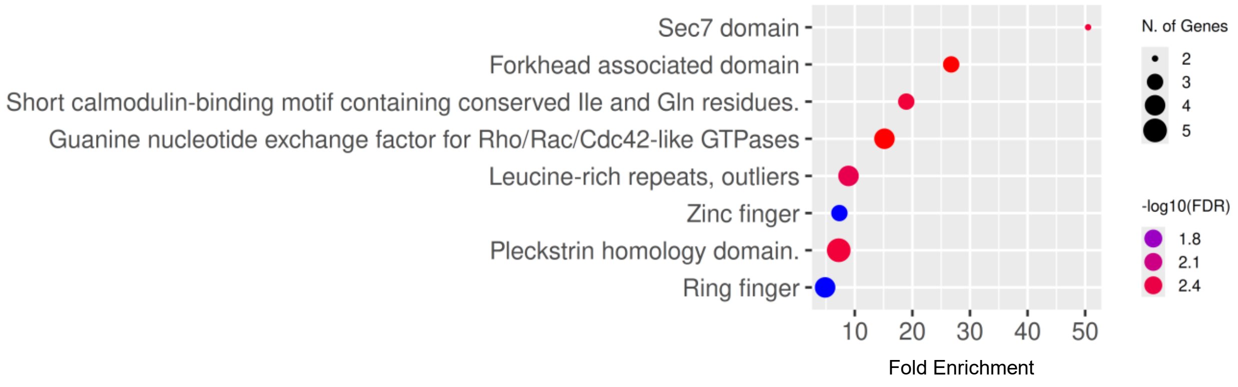

Supplementary Figure S9. Functional enrichment of phosphoproteins whose levels are regulated by AprA at 60 minutes. (**A**) Top enriched pathways, (**B**) GO: Molecular Function, (**C**) SMART protein domains. Bubbles show fold enrichment (x-axis); bubble size represents the number of genes; bubble color indicates statistical significance as –log10(FDR), ranging from blue (minimum) to red (maximum). FDR cutoff = 0.05, pathway size = 5–1000.

Supplementary Figure S10: Functional enrichment of PolyP-regulated phosphoproteins at 60 minutes

A: Top enriched pathways

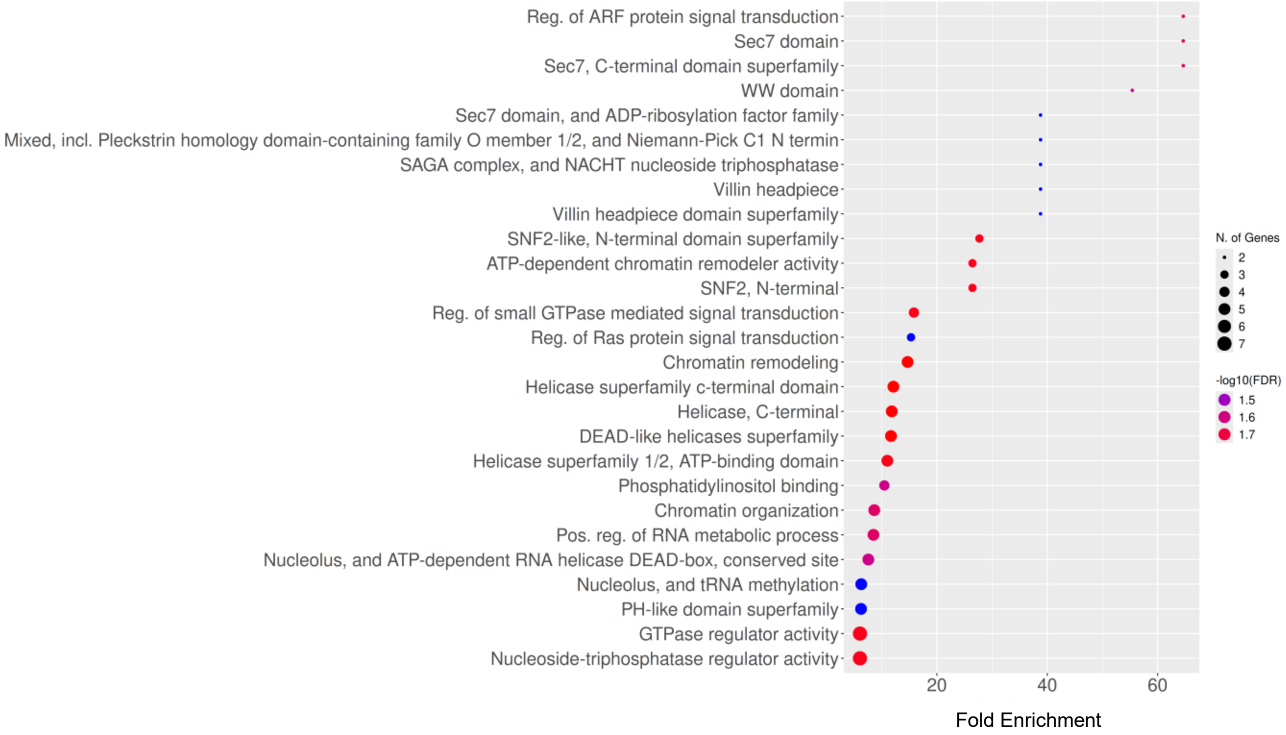

B: GO: Molecular functions

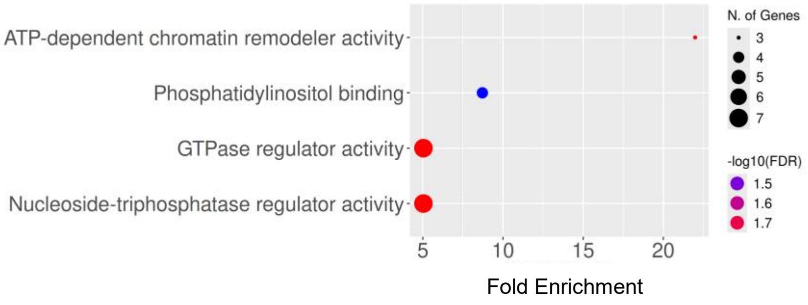

C: GO: Biological Process

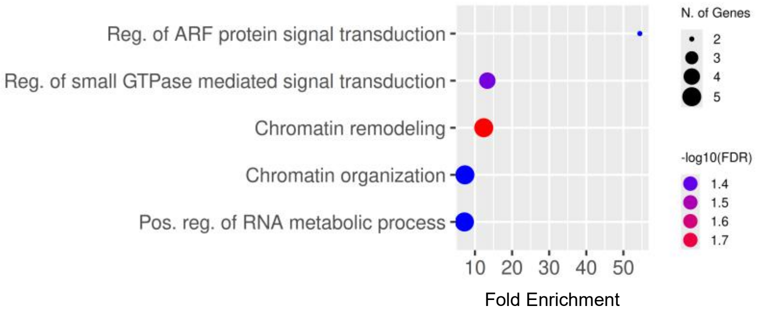

D: Protein domains (SMART)

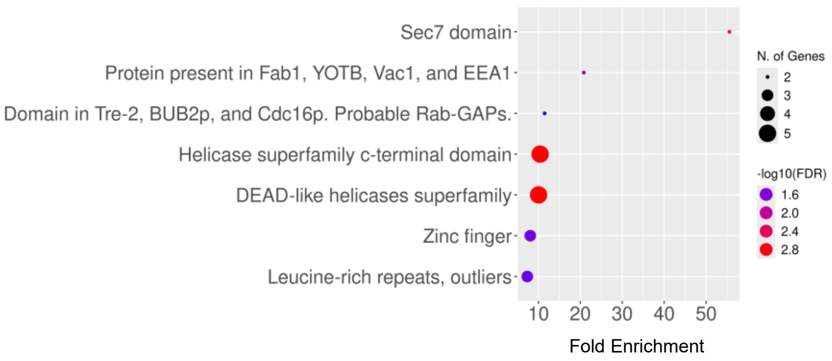

Supplementary Figure S10. Functional enrichment of phosphoproteins whose levels are regulated by polyP at 60 minutes. (**A**) Top enriched pathways, (**B**) GO: Molecular Function, (**C**) GO: Biological Process, and (**D**) SMART protein domains. Bubbles show fold enrichment (x-axis); bubble size represents the number of genes; bubble color indicates statistical significance as –log10(FDR), ranging from blue (minimum) to red (maximum). FDR cutoff = 0.05, pathway size = 5–1000.

Supplementary Figure S11: Chemotactic responses of phosphoprotein mutants in AprA and polyP gradients

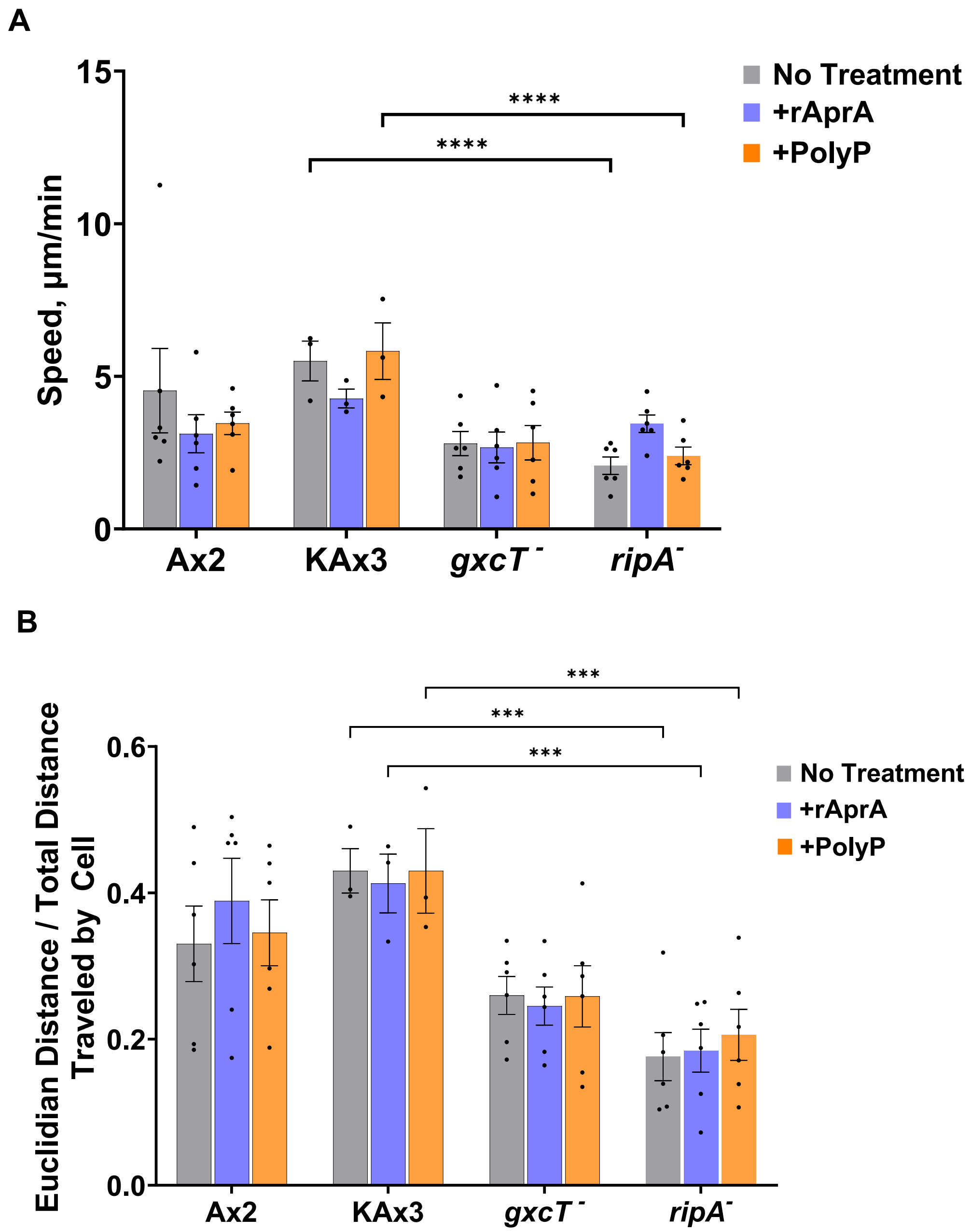

Supplementary Figure S11. Chemotactic responses of phosphoprotein mutants in AprA and polyP gradients. (**A**) Average migration speed of the indicated strains. Bars are mean ± SEM, $n \geq 3$. (**B**) Directional persistence of cell movement trajectories. Bars are mean ± SEM, $n = 6$. *** $p < 0.001$, **** $p < 0.0001$ (Two-way ANOVA with Tukey's multiple comparisons test among three different groups within respective parental and mutant).
